# In-Situ Thermometry Reveals Fragmentation Behavior Based on Local Temperature in α-Olefin Polymerization Catalysts

**DOI:** 10.1021/jacs.4c11357

**Published:** 2025-02-10

**Authors:** Joren M. Dorresteijn, Bas Terlingen, Koen W. Bossers, Thimo S. Jacobs, Yevkeni Wisse, Peter de Peinder, Virginie Cirriez, Alexandre Welle, Eelco T. C. Vogt, Florian Meirer, Bert M. Weckhuysen

**Affiliations:** † Inorganic Chemistry and Catalysis Group, Debye Institute of Nanomaterials Science and Institute for Circular and Sustainable Chemistry, Utrecht University, Utrecht 3584, CG, The Netherlands; ‡ VibSpec, Haaftenlaan 28, 4006 XL Tiel, The Netherlands; § R&D Polymer Differentiation Team, TotalEnergies One Tech, Zone Industrielle C, 7181 Feluy, Belgium

## Abstract

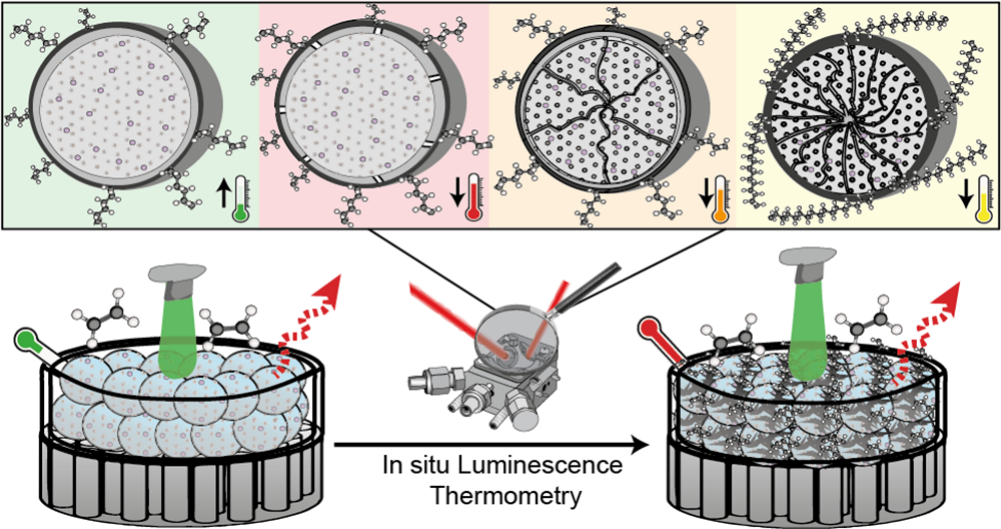

Typical industrial olefin polymerization processes to produce both commodity and specialty polyolefin grades are mainly based on spherical, heterogeneous catalyst particles. During α-polymerization, heat from the exothermic reaction and pressure induced by the growing polymer chains on the catalyst particle lead to fragmentation, revealing active sites for further polymerization. To study these phenomena precisely and in-depth, we utilized a Nd-doped LaOCl-supported metallocene model system. This model catalyst can accurately display fluctuations in temperature with luminescence thermometry. During mild gas phase prepolymerization conditions, we observed a temperature difference of +43 °C and link this to the exothermicity of the ethylene polymerization reaction. In addition, the fragmentation behavior of the model catalyst was accurately monitored. The shell feature of the catalyst ruptured layer-by-layer, while the inner core fragmented via a bisectional fragmentation mechanism. We demonstrated that it is possible to probe the individual temperatures of multiple catalyst support particles within the field-of-view of the probe. This was correlated to structural changes and kinetics in an α-olefin polymerization catalyst. This powerful toolbox could be applied to different heterogeneous catalytic systems to correlate the temperature profile with morphological evolution.

## 1 Introduction

The use of plastics is deeply integrated into our daily lives. In 2021, the global polyethylene (PE) market alone was estimated to be a 110 billion dollar industry, and it is expected to grow to 142 billion dollars by the end of 2029.^1,2^ PE is a polyolefin containing only hydrogen and carbon, making it an energy-effective and sustainable product, once it can be made from renewable resources and properly recycled.^3^ Currently, different grades of PE (such as high-density PE (HDPE), ultrahigh molecular weight PE (UHMwPE) and low linear density polyethylene (LLDPE)) are industrially produced by mainly heterogeneous catalysts. Commonly used heterogeneous catalysts are Ziegler–Natta (TiCl_4_ on MgCl_2_ or MgCl_2_/SiO_2_ activated by trialkylaluminum species), Phillips (CrO_*x*_ on SiO_2_) or single-site catalysts (e.g., Zr or HfCl_2_X_2_, Zr or HfMe_2_X_2_ species on SiO_2_ activated by methylaluminoxane (MAO)) systems.^4^ The physicochemical properties of PE can be tailored by altering the degree of short- and long-chain branching, the overall molecular weight and the molecular weight distribution.^5^ The resulting distinct characteristics arise from these intrinsic molecular arrangements. This makes a PE polymer broadly used from packaging and construction to medical industries and protective equipment.^6^ To obtain and maintain high-quality PE products and ensure continuous operation at the kiloton scale industrial reactor, advanced insights are required especially for state-of-the-art immobilized single-site catalyst systems.^7^

One of the key elements and most researched phenomena since 1970 is the fragmentation behavior of the α-olefin polymerization catalyst.^8^ Fragmentation is the process of the α-olefin polymerization catalyst fracturing due to stress and temperature build-up because of polymer formation on the catalyst inner pores and cavities and outer surfaces.^9^ This process is essential to sustain catalyst activity as it reveals additional active sites and lowers mass transfer barriers for polymerization, even when a dense polymer layer has surrounded the exterior catalyst surface.^10^ Fragmentation behavior is therefore a complex interplay between physical and chemical catalyst properties, ranging from pore size and mechanical strength to MAO and active site weight loading to the distribution of the these species.^11,12^ Typically, fragmentation behavior is described by a combination of two limiting or fundamental modes.^9,13,14^ The first limiting mode is called layer-by-layer, also known as the shrinking core, which can be described by peeling off the outer surface catalyst layer propagating to the inner core. The second limiting mode, continuous bisection, cleaves to the particle’s inner core and progresses by fracturing internally into smaller pieces.

Previous modeling and experimental work on this fragmentation behavior has encompassed a wide range of different reaction conditions,^14−16^ thermal effects,^17−19^ model structures,^20−22^ catalyst systems,^23−25^ high-resolution 3D imaging^26−29^ and supports.^30−32^ However, a powerful analytical toolbox has not yet been reported for elucidating the fragmentation behavior based on local temperature changes inside the catalyst particle for α-olefin polymerization catalysis.^33^

To realize this, we report the follow-up of the Ziegler lanthanum oxychloride (LaOCl) spherical cap model catalyst:^21^ a metallocene-based α-olefin polymerization spherical Nd:LaOCl catalyst. It is expected that there is a difference in terms of surface chemistry between LaOCl and conventional SiO_2_ supports when they interact with MAO. However, this model system provides three main advantages. First, the local temperature of the entire catalyst volume can be accurately measured based on luminescence thermometry as the framework consists of LaOCl doped with 2% Neodymium, enabling a study of local exothermicity of the polymerization reaction during operation. Second, La provides strong imaging contrast, for instance, in the backscattered electron mode of scanning electron microscopy (SEM) or X-ray based imaging techniques because of its high atomic weight. This facilitates excellent separation of phases when studying the early fragmentation kinetics of the catalyst. Last, the high mesoporosity and a shell feature of the Nd:LaOCl microspheres induced by the templating ultrasonic spray pyrolysis method mimic the structural features that industrial α-olefin polymerization catalysts possess.^31^ These features make our model system relevant for industrial catalysts.

## 2 Results and Discussion

### 2.1 Formation and Operation of the Catalyst

**Figure 1:**
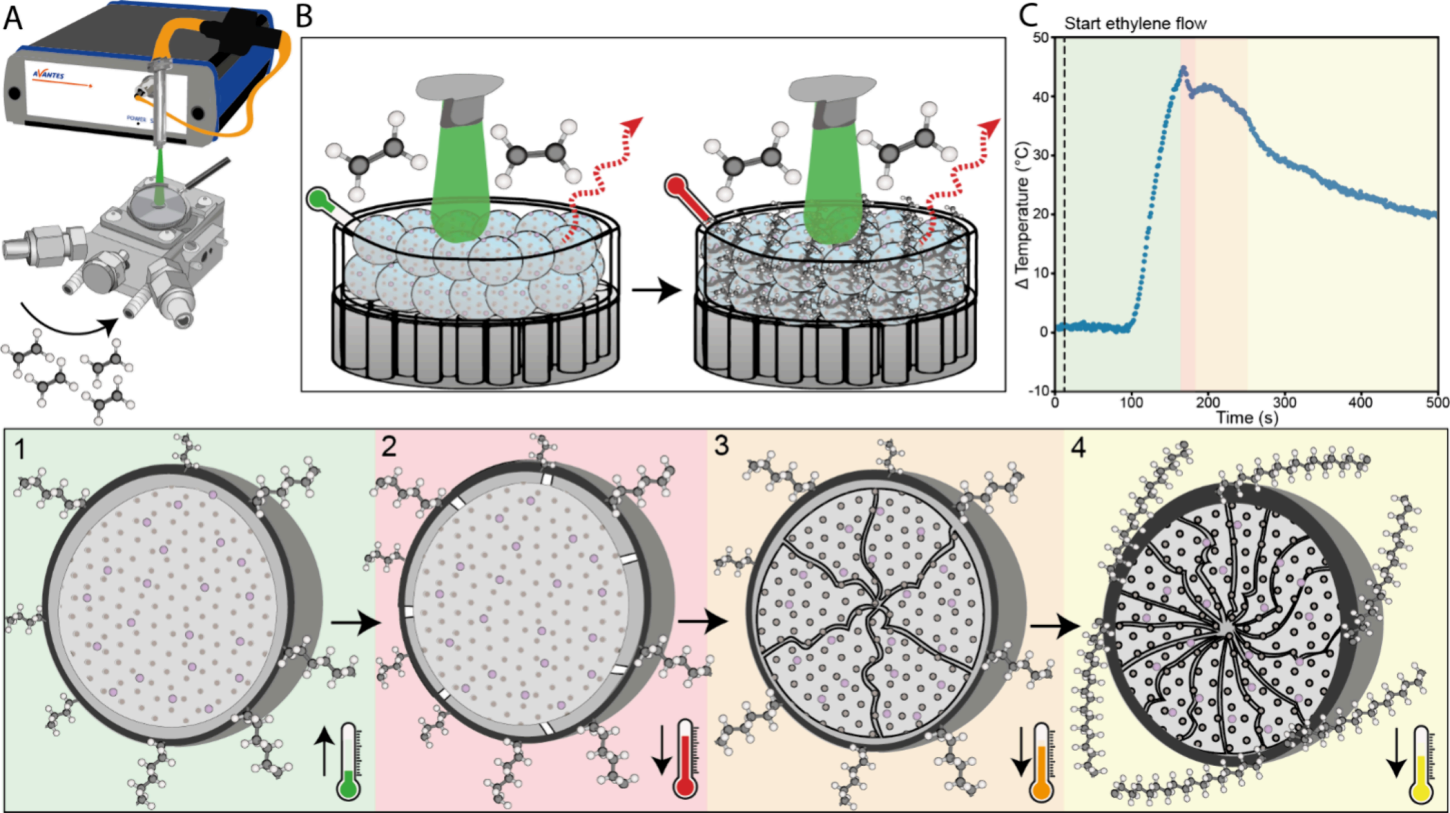
Schematic overview of the Nd:LaOCl/MAO/ZrCp_2_Me_2_ α-olefin polymerization catalyst during the ethylene gas-phase polymerization reaction. (A) Operation of the catalyst during ethylene gas-phase polymerization while loaded in a Harrick cell and equipped with a 532 nm fiber-coupled optical probe. (B) Nd-LaOCl catalysts during catalysis emitting infrared light based on the local catalyst temperature. (C) In-situ luminescence thermometry measurement, showing the temperature profile of the Nd:LaOCl/MAO/ZrCp_2_Me_2_ catalyst during the first 500 s of a 60 min gas-phase ethylene polymerization experiment. Polymerization regimes indicated by color.

We investigated a Nd:LaOCl supported zirconocene-based catalyst (Nd:LaOCl/MAO/ZrCp_2_Me_2_). When the catalyst is excited by a 532 nm laser during the ethylene gas-phase polymerization experiments in the Harrick cell (Figure 1A), the catalyst exhibits a temperature-sensitive luminescence. The supporting matrix, ∼1 μm microspheroidal LaOCl doped with 2% neodymium, was synthesized by a templating ultrasonic spray pyrolysis procedure (SI, section 1) and consisted of a tetragonal *P*4/*nmm* crystal structure with successive layers of La^3+^ or Nd^3+^ cations and Cl^–^ or O^2–^ anions (Figure S2, as confirmed by XRD, Figure S20).^21^ After initial synthesis of the Nd:LaOCl support, the support was activated with MAO, followed by impregnation with the Cp_2_ZrMe_2_ metallocene (SI, 1b). This yielded a final active catalyst for ethylene polymerization with a composition comprising Nd loading of 1.4 wt %, Al loading of 20 wt %, and Zr loading of 0.7 wt % confirmed by ICP-OES (SI section 4e). The Al/Zr ratio of 28 is however nonideal for metallocene catalysts, as metallocene catalysts operate better with higher Al/Zr ratios of around 100. Effects of different synthesis conditions have also been studied for the LaOCl microspheres, in terms of improving the physical parameters. This was specifically done for surface area and pore volume and can be found in SI section 5.

The structure of the final catalyst was further analyzed with N_2_ physisorption and (FIB)-SEM-EDX to address physical properties, morphology, pore structure, and alumina distribution. Cross sections of multiple catalyst particles confirmed that the catalyst had a spherical size between 0.5 and 3 μm and possessed a homogeneous mesoporous structure with a shell feature (Figure S5), in contrast to Nd:LaOCl produced without a template (Figure S6). This mimics the morphology of conventional α-olefin polymerization silica supports.^34^ Alumina from MAO was homogeneously distributed over the catalyst according to SEM-EDX (SI, section 4c). N_2_ physisorption measurements of Nd:LaOCl support, Nd:LaOCl/MAO and Nd:LaOCl/MAO/Cp_2_ZrMe_2_ showed that MAO grafted into the pores of the Nd:LaOCl support was indicated by a decrease in average pore size and preferential grafting into the larger pores of the support (SI, section 4d). After metallocene impregnation, a further decrease of pore volume and surface area was observed, indicating anchoring of the metallocene into the pores of the metallocene onto the MAO (SI, section 4d), and was confirmed by ICP-OES (SI, section 4e).

Loosely bound MAO species were not found in the activated sample or catalyst since a peak at ∼4 nm was only observed in BJH desorption, not in BJH adsorption (SI, section 4d).^34^ This implies that the 4 nm peak is a phantom peak caused by the tensile strength effect.^35^ Besides, heterogeneity in anchoring sites also is expected because of the Lewis acidic nature of the LaOCl framework, which could give rise to different metallocene anchoring sites.^36,37^

When the catalyst is excited by a 532 nm laser (Figure 1B), sharp emission lines are yielded which can be assigned according to the Dieke diagram of Nd^3+^ (Figure S3A).^38−41^ Temperature dependent emission is measured between 25 and 100 °C of the catalyst. Variations in relative emissions are observed between the thermally coupled ^4^F_5/2_ and ^4^F_3/2_ levels (Figure S3B). The emission from these energy levels to the ground state was measured at different temperatures, both before and after polymerization, under inert N_2_ flow conditions (Figure S3C). The natural logarithm of the ratio of these two emission regions versus the reciprocal temperature yielded a straight line, according to the Boltzmann equation (SI, section 4a).

### 2.2 Exothermicity and Fragmentation Behavior of the Nd:LaOCl/MAO/Cp_2_ZrMe_2_ Catalyst

After the initial temperature calibrations of the catalyst system (SI, section 4a), in situ gas-phase ethylene polymerization experiments (SI, section 3) under very mild conditions without external heating were performed to address the local temperature changes in the catalyst during operation. At first, a long ethylene gas-phase polymerization experiment (without H_2_) of 60 min at room temperature and 1.2 bar ethylene pressure was carried out to study the full kinetic profile of the catalyst (First 500 s: Figure 1C, full curve in Figure S4). The total temperature change of the reaction was found to be 43 °C, while the total reaction could be divided into four temperature regimes. This was further studied with CO_2_ quenching experiments to instantly stop the reaction. These experiments were carried out to pinpoint the exact moment of physical change in the catalyst during the polymerization reaction, as the induction time of CO_2_ was always ∼10 s.

**Figure 2:**
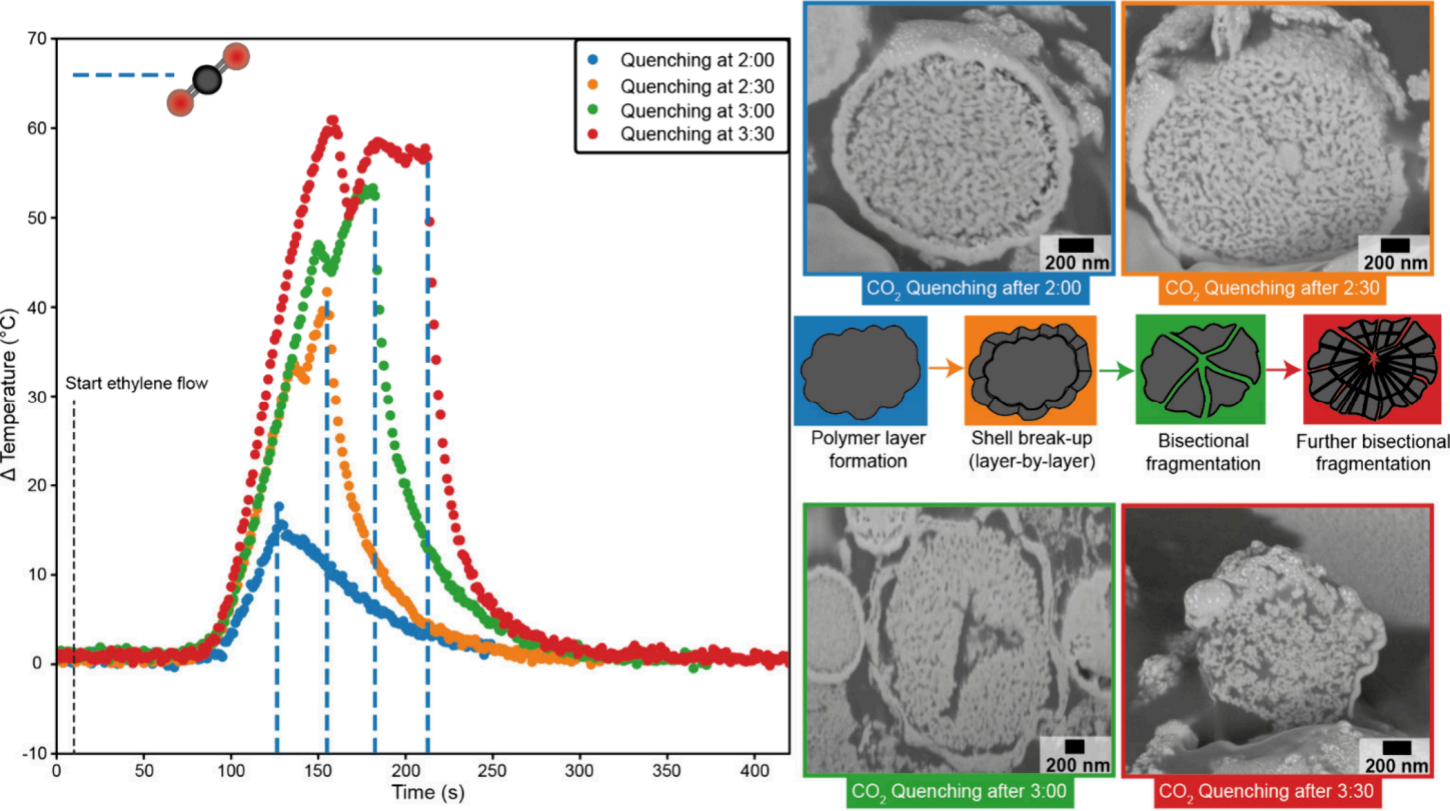
Temperature profile of a Nd:LaOCl/MAO/Cp_2_ZrMe_2_ olefin polymerization catalyst during the ethylene gas-phase polymerization reaction in the prepolymerization phase. The blue dotted lines indicate the moment the catalyst was poisoned with CO_2_, showing an immediate decrease in temperature and activity. The quenched samples were subsequently analyzed with FIB-SEM to visualize the structural changes in the catalyst material. In the schematic, a representation of the different phases of ethylene polymerization is shown. Blue, indicating polymer layer formation surrounding the catalyst particle. Orange, layer-by-layer fragmentation in the catalyst shell structure. Green, bisectional fragmentation occurring in the center of the catalyst particle. Red, bisectional fragmentation occurring throughout the whole catalyst particle.

The reaction starts with an induction period from *t* = 0 to 145 s (Figure 2, color code: blue), with a PE shell being formed, while the temperature increases rapidly because of the exothermicity of the reaction. Then, the reaction reaches the maximum temperature and proceeds to start with layer-by-layer fragmentation from *t* = 145 to 168 s (Figure 2, color code: orange), rupturing the shell of the catalyst. Next, the full fragmentation stage initiates from *t* = 169 to 210 s (Figure 2, color code: green), with the catalyst inner core starting to fragment bisectional. Finally, the reaction ends with a full fragmentation and particle expansion steady state, which shows a slow temperature decay propagating from *t* = 211 to 3600 s (Figure 2, color code: red), whereby the catalyst completely fragments bisectionally and the polymer fully expands over the catalyst. The polymerization curves of each polymerization (Figure 2) show the same behavior, although differences in total temperature change can be observed at up to 60 °C. This indicates that even in the same batch of produced catalysts there is a detectable heterogeneity in morphology and presumably packing of the catalyst particles that result in a difference of total Δ*T*. Subsequently, this difference in total Δ*T* between the reactions is caused by differences in heat transport within the cell. Note that the cell used here does not accurately represent the conditions in an industrial gas-phase type reactor.

**Figure 3:**
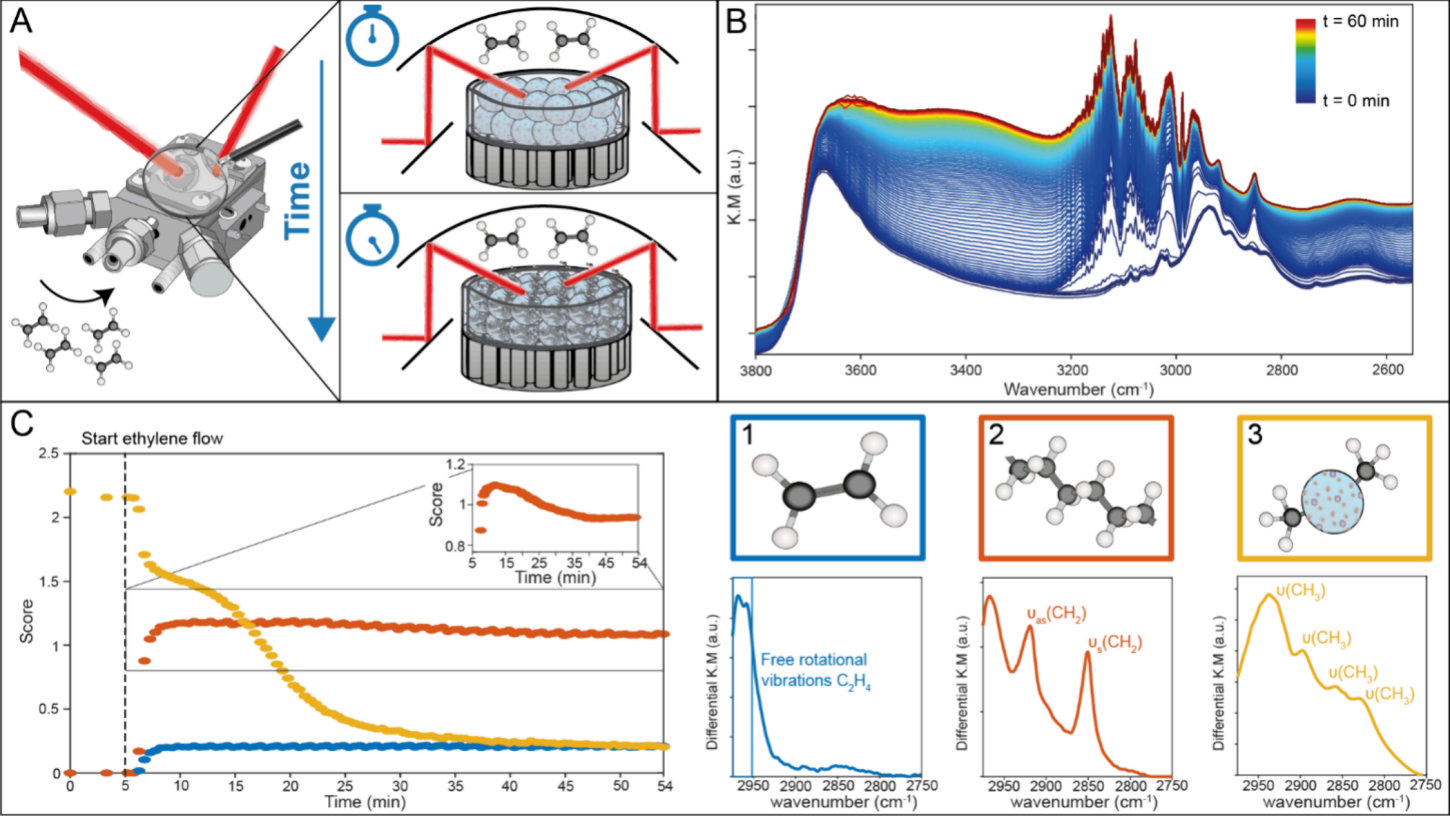
Nd:LaOCl/MAO/Cp_2_ZrMe_2_ catalyst during the ethylene gas-phase polymerization reaction, as measured with DRIFTS. (A) Schematic of the DRIFTS cell during operation. (B) Time evolution of the DRIFTS preprocessed spectra of the Nd:LaOCl/MAO/Cp_2_ZrMe_2_ catalyst during 60 min ethylene polymerization. Dark blue is spectrum 1, red spectrum 120. (C) Results of PCA and subsequent MCR of the measured DRIFTS spectra displaying the evolution of three different components (Component 1, blue: ethylene gas, Component 2, red: PE, Component 3, yellow: catalyst) over time; the last 6 min of the experiment have been removed because of oversaturation.

After interpretation of the four main reaction regimes in the ethylene polymerization, diffuse reflectance infrared Fourier transform spectroscopy (DRIFTS) of the ethylene polymerization process was performed under similar reaction conditions for comparison with the in situ thermometry polymerization. The DRIFTS experiment (Figure 3A) was carried out with lower catalyst loading (13 mg vs 26 mg) to prevent signal oversaturation when using the same reaction time; however, signal oversaturation still occurs after 50 min reaction time (Figure 3B). Principal component analysis (PCA) and subsequent multivariate curve resolution (MCR) were performed on the DRIFTS data series (Figure 3C), revealing that during operation the system consists of three phase changes.

Component 1 depicts the ethylene gas during the reaction (Figure 3C, color code: blue), recognizable by the free rotational vibrations between 2975 cm^–1^ and 2950 cm^–1^. The profile of component 1, ethylene, shows a steady increase from *t* = 6.5 to 8.5 min, displaying the start of the flow over the catalyst bed, with the flow reaching a steady state with periodic fluctuation every 1.5 min, showing a small inconsistency with the mass flow controller. Component 2 (Figure 3C, color code: red) indicates the growth of PE, observable from the υ_as_(CH_2_) at 2920 cm^–1^ and υ_s_(CH_2_) at 2851 cm^–1^ peaks. The component 2 score plot resembles the kinetic profile of a metallocene-based silica-supported catalyst to a certain extent and shows a similarly fast increase in PE formation to the temperature rise in the in situ thermometry experiment.^42^ Interestingly, the PE formation displays a fluctuation similar to the ethylene flow, indicating that both phases are still mixed in the MCR. The thermal effects, as the polymer formation releases heat, cause severe band shifts, making unmixing of the different phases more difficult.^43,44^ However, the profile can be described in a fashion very similar to that of the in situ thermometry profile. The profile starts with the fast growth of the polymer layer enclosing the catalyst particles, followed by an induction period characterized by mass-transfer limitations and finally the fragmentation and steady-state phase. The latter stages show a slowly decaying profile. Unfortunately, as the time resolution is not high enough (1 spectra every 30 s), the fragmentation of the outer layer is not visible. Component 3 (Figure 3C, color code: yellow) resembles the catalyst phase, consisting of four methyl bands. These four bands at 2940, 2898, 2859, and 2830 cm^–1^ depict methyl groups, present in the active site (Cp_2_ZrMe_2_) and cocatalyst (MAO). The component 3 score plot indicates the inverse of the PE formation at the start, showing the highest score at the start when the catalyst is still intact. After insertions into the CH_3_-bond of the metallocene during initial ethylene flow, polymer grows surrounding the particle, showing a vast decrease of component 3. Then, the catalyst phase slowly decreases during the fragmentation stage, as the CH_3_/CH_2_ or methyl to polymer ratio decreases as higher molecular weight PE is forming. After this decay, the profile shows another decay until the catalyst phase resembles a score close to zero as the catalyst phase is covered in PE and cannot be probed anymore by IR. The scores of the components therefore indicate the concentration of different phases. However, differences in light path, i.e., path length and scattering effects, due to PE formation complicate the preciseness of the actual concentration. It can be observed that the total concentration represented by the total score (consisting of the three phases, ethylene, polyethylene, and catalyst) changes over time. From a 2.25 total score at *t* = 0 min and the highest total score of 3 at around *t* = 7 min and a total score of 1.6 at *t* = 54 min, this change in total score can be explained by the change of the different phase composition, which has a different penetration depth through the different phases, as the penetration depth through ethylene gas or amorphous PE is much further than through a dense catalyst LaOCl/MAO/Zr phase.^45,46^ This is accompanied by the penetration depth of the laser, which only probes part of the surface and is also wavelength dependent, which is theoretically around 2 μm at a wavenumber of 2000 cm^–1^ and around 500 nm at a wavenumber of 4000 cm^–1^. Both these factors complicate the accurate representation of the total concentration; however, even with a PE score mildly decreasing over time, it can be observed that it is still polymerizing. First, the relative score of the polymer phase in comparison to the total score significantly increases over time, as the catalyst score between min 7 to 45 decreases in a 3-step regime from 1.6 to 0.3, and the ethylene score is stable, meaning that the PE phase score relative to the total concentration of phases increases. Therefore, this shows that the catalyst concentration significantly decreases over time, meaning breakup of larger catalyst fragments to smaller pieces and growth of polymer (even if the PE score goes from ∼1.05/1.1 to ∼0.95). The small decrease in PE concentration can also be explained by the fact that DRIFTS only probes the top part of the sample and how far the polymerization progresses. Less catalyst and therefore active sites will be in this top part of the sample. Furthermore, it is also possible that the PE score can decrease due to swelling phenomena as there is more dissolved ethylene in this layer over time.^47−49^

The resemblance between the in situ thermometry and DRIFTS results therefore confirms the kinetic models in available literature.^11,12,42^ However, heat convection in the cell and a stagnant catalyst bed in the early stages of polymerization should be addressed for further applicability of in situ thermometry in α-olefin polymerization catalysis. Finally, it should be mentioned that extrapolation to industrially relevant systems of typically 20–70 μm in size of either MgCl_2_ and SiO_2_ could currently not be provided and would require further optimization of both the catalyst system and reactor cell.

## 3 Conclusions

The strength of in situ thermometry for α-olefin polymerization catalysis has been explored by investigating the fragmentation behavior and polymerization kinetics of a Nd:LaOCl/MAO/ZrCp_2_Me_2_ ethylene polymerization catalyst. The ethylene gas-phase polymerization under very mild conditions could be investigated during the highly exothermic early polymerization phase with 50 ms precision. The model system showcased in this study closely resembled the features of an industrial gas-phase metallocene polymerization catalyst. However, the catalyst displayed an unoptimized polymerization behavior. This was mainly because of factors concerning heterogeneity in active sites stemming from a low Al/Zr ratio of 28 and particle overheating during operation because of low heat convection. Both synthesis and analytical toolboxes showcased here have direct applicability to different α-olefin polymerization catalyst types, as the model support closely resembles polymerization carriers and shows similar kinetic behavior as silica and therefore can be applied for different metallocene active sites and Ziegler(-Natta) type catalysts. Therefore, we showcased a broad applicable model system that could accurately probe the catalyst temperature and correlate this to structural changes and kinetics in an α-olefin polymerization catalyst, which brings the elucidation of polymerization fragmentation one step closer. This could also be applied for different heterogeneous catalytic reactions of both exo- and endothermic, for example, CO_2_ hydrogenation, dry reforming of methane, or methanol-to-olefin reactions. Future improvements would include single particle luminescence studies on a diluted catalyst bed to study intraparticle heterogeneities linked to the fragmentation behavior and kinetic profiles.

## Supporting Information Available

Experimental methods and characterization, methodology for data analysis, setup configurations, and changes of synthesis conditions on LaOCl microspheres (PDF)
