# Supplementary material for: In-Situ Thermometry Reveals Fragmentation Behavior Based on Local Temperature in α-Olefin Polymerization Catalysts
Source: J Am Chem Soc. 2025 Feb 10;147(7):5642–8. doi: 10.1021/jacs.4c11357 (PMC11848817; doi:10.1021/jacs.4c11357)
Supplement: Supplementary file 1 — ja4c11357_si_001.pdf [file ja4c11357_si_001.pdf]

Supporting Information

In-situ Thermometry Reveals Fragmentation Behavior Based on Local Temperature in α-Olefin Polymerization Catalysts

Joren M. Dorresteijn[†], Bas Terlingen[†], Koen W. Bossers[†], Thimo S. Jacobs[†], Yevkeni Wisse[†], Peter de Peinder[†,‡], Virginie Cirriez[Ł], Alexandre Welle[Ł], Eelco T.C Vogt[†], Florian Meirer[†], Bert M. Weckhuysen[†,*]

[†] Inorganic Chemistry & Catalysis group, Debye Institute for Nanomaterials Science and Institute for Sustainable and Circular Chemistry, Utrecht University, 3584 CG Utrecht, The Netherlands
[‡] VibSpec, Haaftenlaan 28, Tiel, The Netherlands.
[Ł] Polymer Differentiation Team R&D, Totalenergies One Tech Belgium, Zone Industrielle C, 7181 Feluy, Belgium
[*] Corresponding Author: Bert Weckhuysen (b.m.weckhuysen@uu.nl)

Table of Contents

1. Experimental methods
    a. Spray pyrolysis of Nd:LaOCl microspheres
    b. Activation of Nd:LaOCl microspheres
    c. In-situ luminescence spectroscopy matrix calibrations
    d. In-situ temperature measurements with CO_2 quenching during gas-phase ethylene polymerizations
    e. In-situ diffuse reflectance infrared Fourier transform spectroscopy during ethylene polymerization
2. Catalyst characterization methods
    a. Focused ion beam milling-scanning electron microscopy
    b. Scanning electron microscopy-energy dispersive X-ray spectroscopy
    c. Inductively coupled plasma-optical emission spectroscopy
    d. N_2 physisorption
    e. X-ray diffraction
    f. Differential scanning calorimetry
3. Set-up configurations
    a. Ultrasonic spray pyrolysis of Nd:LaOCl microspheres
    b. In-situ temperature measurements with CO_2 quenching gas-phase set-up
4. Results – Additional data for different analytical techniques
    a. In-situ thermometry data
    b. Scanning electron microscopy images and cross-sections of supports, catalyst and polymers
    c. Scanning electron microscopy-energy dispersive X-ray spectroscopy of cross sections of catalyst and polymers
    d. N_2 physisorption data
    e. Inductively coupled plasma-optical emission spectroscopy data
    f. X-ray diffraction data
    g. Differential scanning calorimetry data
5. Results - Changes of reaction conditions on LaOCl microspheres
    a. List of Experiments
    b. Effect of CTAB concentration
    c. Effect of carrier gas flowrate
    d. Effect of carrier gas
    e. Effect of temperature
    f. Effect of surfactant (P123)
    g. Particle size distributions all LaOCl experiments
6. References

1. Experimental Methods

1a. Spray Pyrolysis of Nd:LaOCl microspheres

The Nd:LaOCl microspheres were prepared by a ultrasonic spray pyrolysis process consisting of an ultrasonic nebulizer (1.7MHz), a quartz tube (ID = 55 mm and length = 550 mm), an electrical tube furnace (400mm, TZF 12/38/400, Carbolite Gero Ltd.) and 3 washing flasks (2L Laboratory bottle DURAN™).

Lanthanum(III) chloride heptahydrate ($LaCl_3 \cdot 7H_2O$, 99%, Alfa Aesar) (0.27M), Neodymium(III) chloride hydrate ($NdCl_3 \cdot xH_2O$, 99.9%, Alfa Aesar) (0.0054M) and Cetrimonium bromide (CTAB, ≥98%, Sigma-Aldrich) (0.0027M) were used in the spray solution. The spray solution was prepared by dissolving both lanthanum and neodymium salts together with CTAB in demineralized water. The prepared precursor solution was turned into droplets using an ultrasonic nebulizer and carried into the quartz reactor (at 700°C) by an air flow (800 mL/min). The produced microspheres were collected in the 3 washing flasks which were filled in sequential 2L, 1L and 1L 0.1M ammonium hydroxide (28.0-30.0% NH_3 in H_2O, ≥99.99% , Sigma-Aldrich) in ethanol (absolute, VWR) solution.

Microsphere collection was done by washing the white precipitate with ethanol and centrifuging at 4000 rpm for 6 minutes and then vacuum filtrated over a 0.45 µm nylon membrane (GVS S.p.A.). The final product was dried for 2 hours 60 °C in air and then calcined at 550 °C for 5 h (2 °C/min ramp rate) under active air flow. The white powder was then transferred at 200 °C into a glovebox operating at <1 ppm O_2 and H_2O and stored for further activation.

1b. Activation of Nd:LaOCl microspheres

Methylaluminoxane (MAO) impregnation was performed by mixing the Nd:LaOCl microspheres to a 30% wt.% MAO (Albemarle) in toluene for 4h under reflux in an inert atmosphere, with a target loading around 20 wt.% Al, similar to the synthesis procedure of *Zanoni et al.*[1] The obtained Nd:LaOCl supported MAO was then washed extensively with toluene and pentane (both under Ar, Alfa Aesar™, anhydrous, dried over molecular sieves) and vacuum dried. The dry Nd:LaOCl supported MAO was then impregnated with a toluene solution of the metallocene catalyst Cp_2ZrMe_2 (bis(cyclopentadienyl)dimethyl-zirconium(IV) 97%, Sigma Aldrich) for 2 h at room temperature to reach the desired metallocene loading of 0.7 wt%. After impregnation, the catalyst was washed with toluene and pentane and dried under vacuum to obtain dry light grey/yellow powder, indicating that the metallocene is heterogenized onto the support.

1c. In-situ luminescence spectroscopy matrix calibrations

The excitation and emission of neodymium was performed using a fiber-coupled probe, which was not focused. The emission spectra of neodymium for both the calibration and the in situ temperature measurements were collected using a Avantes AvaSpec-2048L StarLine spectrometer with a slit size of 25 µm. Neodymium was excited using an Avantes AvaRaman-532 HERO-EVO, with a laser wavelength of 532 nm and a laser power of 50 mW. All experiments were performed in a Harrick Raman High Temperature Chamber. The calibrations were performed in a temperature range between 25 °C and 100 °C, with steps of 15 °C. The in situ temperature measurements were performed at room temperature and without external temperature control.

1d. In-situ temperature measurements with CO_2 quenching during gas-phase ethylene polymerizations

The catalysis experiments were performed using ethylene gas (4.5 N purity, Linde), and the gas flows were controlled by Bronkhorst mass flow controllers (MFC). All experiments were performed at ambient pressure (ca. 1 bar). To perform the polymerization experiments the Harrick cell was loaded in the glovebox with a small amount of glass wool, a VICI Jour® stainless steel frit and a self-supporting wafer (pressed with a pixie hydraulic pellet press from Pike technologies without using pressure) 7 mm containing ±30 mg catalyst powder. Before each polymerization experiment, the lines were flushed for

10 minutes with N_2. After flushing of the lines, 10 mL/min ethylene flow at 1 bar was flown over the catalyst bed. At the designated quenching time, the ethylene flow was switched via a 2-way valve to a 10 mL/min at 1 bar CO_2 (4.5, Linde) flow poisoning the catalyst and stopping the reaction.[2,3] Then the catalyst was exposed to air when it was settled to room temperature. The data was analyzed using in-house Python code.

1e. In-situ diffuse reflectance infrared Fourier transform spectroscopy ethylene polymerization
Diffuse reflectance infrared Fourier transform spectroscopy (DRIFTS) experiments were performed in-situ using a Bruker Tensor 37 spectrometer, equipped with a nitrogen cooled MCT (mercury cadmium telluride) detector, and a Harrick Praying Mantis™ High Temperature Reaction Chamber. For each measurement, the sample cup of the reaction chamber was loaded inside a nitrogen glovebox with a small amount of glass wool, a VICI Jour® stainless steel frit and 30 mg of the catalyst. For each experimental run, the loaded reaction cell was transferred to the spectrometer and connected to the gas lines. All experiments were performed in gas-phase at room temperature using an ethylene flow of 10 mL/min at 1 bar. To avoid contamination and deactivation of the sample, the gas lines were flushed with nitrogen for 10 min before introducing ethylene to the reaction cell. FT-IR spectra were recorded in 30 s intervals in the spectral range of 900 - 4000 cm^{-1} with a 4 cm^{-1} resolution and 16 s scan time. MCR was applied in MatLab 9.7 (MathWorks) using the PLS-Toolbox 8.8 (Eigenvector Research). MCR-ALS is a modelling technique that enables the incorporation of system knowledge to guide fitting solutions. Non-negativity constraints were applied and the concentration of ethylene gas and product were set to 0 before the gas introduction. Since the pre-processing for MCR is limited due to the non-negativity constrains, PCA was first applied to estimate the ethylene gas concentration from the spectra. As a pre-processing step the 1st derivative of the spectra was used to mitigate the baseline effects introduced due to the physical changes of the sample. From the scores describing the ethylene gas concentration and the corresponding spectra an experimental ethylene gas spectrum was calculated and used as input for the MCR components. The normalized scores were used as an estimate for the concentration profile. Additionally, the initial spectrum of the pristine catalyst was used as input, that is, a spectral component.

2. <u>Catalyst characterization</u>

2a. Focused Ion Beam Milling-Scanning Electron Microscopy
Focused Ion Beam Milling Scanning Electron Microscopy (FIB-SEM) was performed on a FEI Helios NanoLab G3 UC microscope. Samples were loaded on Al stubs with carbon tape and sputter-coated with 10 nm of Pt, before the measurement. Beam currents of 0.1 nA and 2 kV were used to image particles with dwell times varying between 1 and 5 μs, depending on imaging mode. External morphologies were imaged by collecting secondary electrons (SE) with an Everhart–Thornley detector (ETD), while cross-sections were imaged by collecting back scattered electrons (BSE) with a through-the-lens detector (TLD) with a dual scanning electron microscope focused-ion beam. Cross-sections were obtained with a Gallium FIB, removing half of the particle material and then cleaning with precision milling the exposed cross-section. Sufficiently isolated particles were selected for cross-section analysis based on the observation of a wide range of particles across the sample batch to choose representative particles for each batch. Micrograms are measured by collecting back-scattered electrons (BSE); hence the contrast difference will depend on the atomic number (Z) of the elements constituting the material they have interacted with. The main constituents of catalyst and polymer are La and C, respectively; therefore, these two materials will show different BSE intensities resulting in a greyscale SEM image with high contrast between the two phases.

2b. Scanning Electron Microscopy-Energy Dispersive X-ray Spectroscopy
Scanning electron microscopy-energy dispersive X-ray spectroscopy (SEM-EDX) was performed on FEI Helios NanoLab G3 UC microscope at 10 keV and 0.1 nA. Samples were prepared by putting the LaOCl-Nd polymers in ethanol (absolute, VWR) and putting a droplet of the solution on silicon wafers.

2c. Inductively Coupled Plasma-Optical Emission Spectroscopy
Inductively Coupled Plasma-Optical Emission Spectroscopy (ICP-OES) was performed on a SPECTRO CIROSCCD instrument of SPECTRO Analytical Instruments for determining the chemical

composition. The samples were prepared by aqua regia and measured for the total content of La, Nd, Al and Zr.

2d. N_2 Physisorption

N_2 physisorption measurements of the catalyst materials were performed using a Micromeritics TriStar 3000 instrument operating at −196 °C. Before performing the measurements, the Nd:LaOCl pristine samples were dried for 15h at 300 °C under vacuum. The final catalysts and Nd:LaOCl supported MAO samples were loaded for physisorption measurements inside the glovebox, without a prior drying procedure.

2e. X-ray Diffraction

X-ray diffraction (XRD) measurements were carried out in the range of 10 to 80 °using a step size of 0.5 °and scan speed of 1 s on a Bruker AXS® D2 Phaser diffractometer. The samples were irradiated with Co $K_{\alpha1,2}$ radiation (λ = 1.79026 Å) source.

2f. Differential Scanning Calorimetry

Differential Scanning Calorimetry (DSC) was performed on the polymerized samples using a DSC Q2000 instrument by TA Instruments. Calibration was done with indium and using T zero mode. Peak crystallization temperature (T_c), peak melting temperature (T_m) and heat of fusion (ΔH) were measured. The polymer analysis was performed with a 2 to 10 mg of polymer sample. The sample was first equilibrated at 30 °C and subsequently heated to 220 °C using a heating rate of 50 °C/min (first heat). The sample was held at 220 °C for 5 min to erase any prior thermal and crystallization history. The sample was subsequently cooled down to 0 °C with a constant cooling rate of 10 °C/min (first cool). The sample was held isothermal at 0 °C for 5 min before being heated to 220 °C at a constant heating rate of 10 °C/min (second heat). The endothermic peak of melting (second heat) was analyzed using the TA Universal Analysis software and the peak melting temperature (T_m) corresponding to 10 °C/min heating rate was determined.

3. Set-up Configurations

3a. Ultrasonic spray pyrolysis set-up

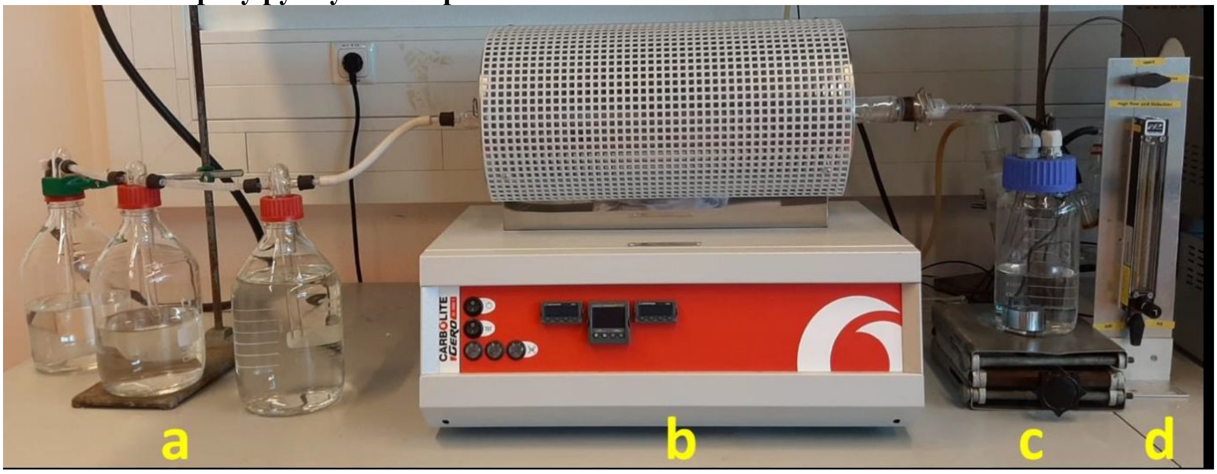

Figure S1: Picture of the ultrasonic spray pyrolysis set-just before operation. During USP experiments, the carrier gas flowrate is determined through (d) a variable flowmeter. The carrier gas transports the precursor droplets, produced by the ultrasonic atomizer into (c) reaction vessel. Then it goes into (b) a tubular oven, the heating zone. Hereafter, the solvent will evaporate and product formation will occur. Subsequently, the product is transported into the product capturing system, (a). The washing flasks. These were filled with a NH_4OH ethanol solution to collect the final product.

3b. In-situ temperature measurements with CO_2 quenching gas-phase set-up

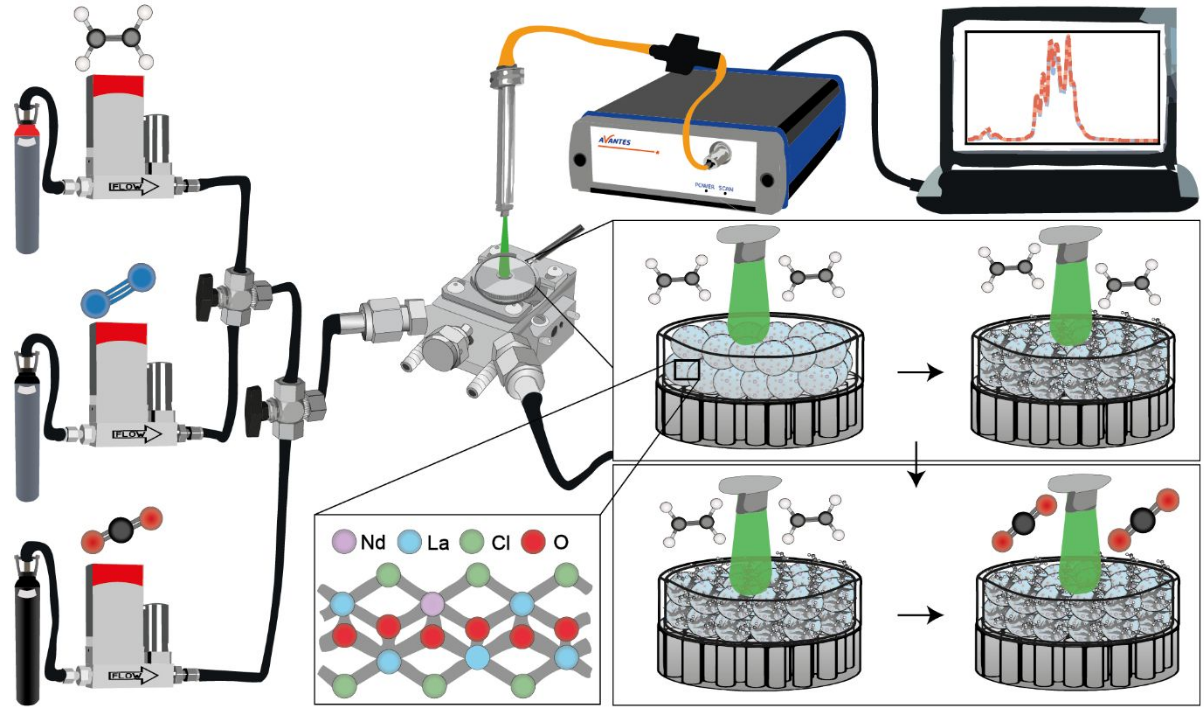

Figure S2: Schematic representation of the in-situ thermometry set-up. The gas lines (C_2H_4, N_2 and CO_2) were connected to Bronkhorst mass flow controllers (MFC). The Harrick cell was connected to two three-way valves connected in series, which were connected to the gas bottles. C_2H_4 and N_2 to the first three-way valve, which was connected to the second three-way valve and CO_2 connected together to the inlet of the Harrick cell. A fiber coupled probe is aimed on top of the Raman window of the Harrick cell, onto the catalyst bed. Since the probed spot is in the order of millimeters, it should sufficiently cancel out photonic artefacts.[4,5] The Harrick cell is connected to a Avantes AvaSpec-2048L StarLine spectrometer with a slit size of 25 µm. The catalyst (consisting of a Nd:LaOCl support with tetragonal P4/nmm crystal structure with successive layers of La^{3+} or Nd^{3+} cations and Cl^{-} anions) was excited using an Avantes AvaRaman-532 HERO-EVO, with a laser wavelength of 532 nm and a laser power of 50 mW.

4. Results

4a In-situ thermometry

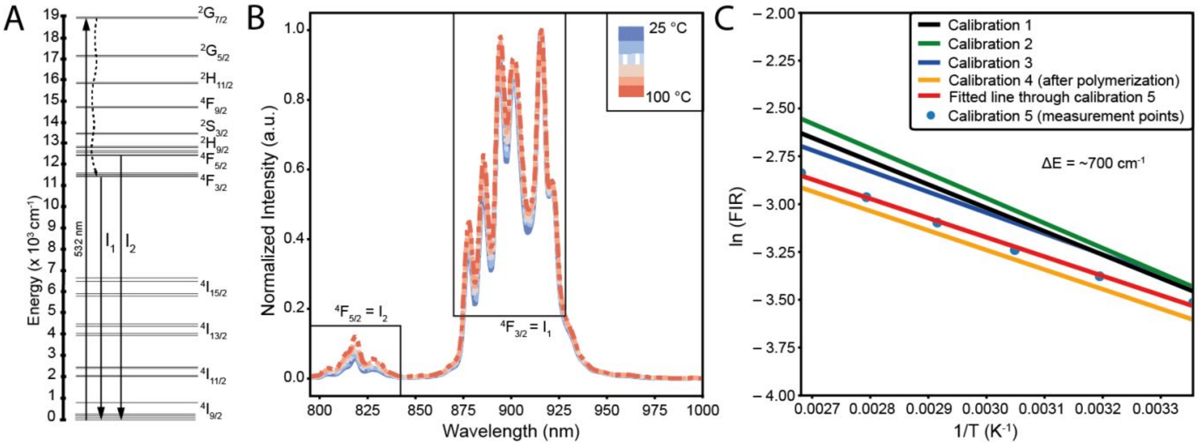

Figure S3. (A) Energy diagram of N^{3+} with indicated energy gap ΔE = 990 cm^{-1} between $^4F_{5/2}$ and $^4F_{3/2}$ states. (B) The emission spectra of Nd:LaOCl support when excited by 532 nm at temperatures between 25 °C (blue) to 100 °C (red), increment of 15 °C. (C) The ratio of integrated emission intensities by indicated $^4F_{5/2}$ and $^4F_{3/2}$ states (I_2/I_1) as a function of the inverse temperature. The straight line is a fit to the Boltzmann model, which yields a $^4F_{3/2}$ - $^4F_{5/2}$ of ΔE = ~700 cm^{-1}. The discrepancy between the calculated value and literature can be described by non-linearity at room temperature, however this does not influence the actual temperature calibration significantly.[6,7]

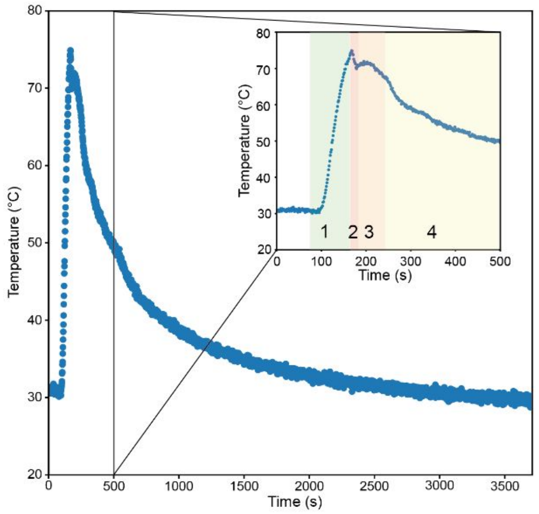

Figure S4: In-situ luminescence thermometry measurement, showing the temperature profile of the Nd:LaOCl/MAO/$ZrCp_2Me_2$ catalyst during a 60 min gas-phase ethylene polymerization experiment, polymerization regimes indicated by color, with the in-set being the first 500s of the experiment.

4b Focused ion beam milling-scanning electron microscopy images and cross-sections of supports, catalyst and polymers

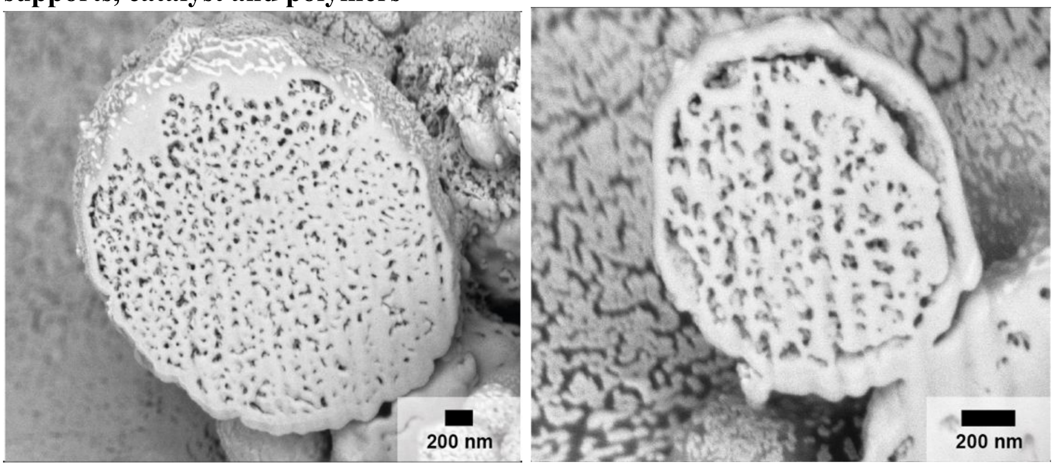

Figure S5: Focused ion beam milling-scanning electron microscopy (FIB-SEM) images of two cross-sections of the LaOCl:Nd support.

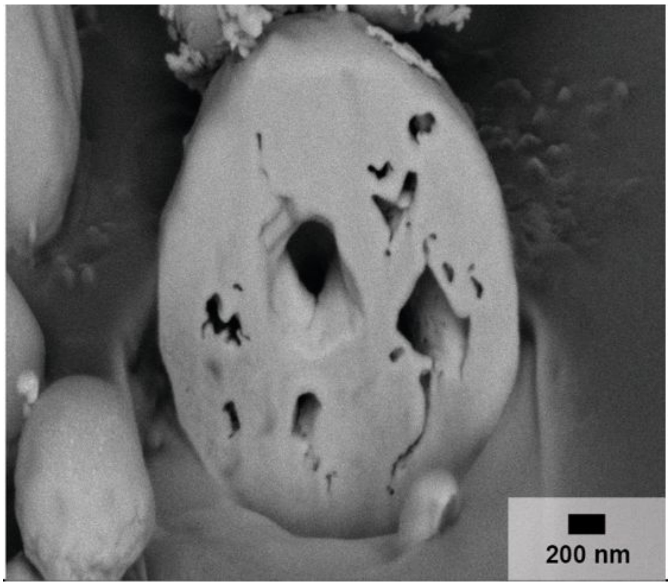

Figure S6: Focused ion beam milling-scanning electron microscopy (FIB-SEM) image of a cross section of the LaOCl synthesis without the use of cetrimonium Bromide (CTAB).

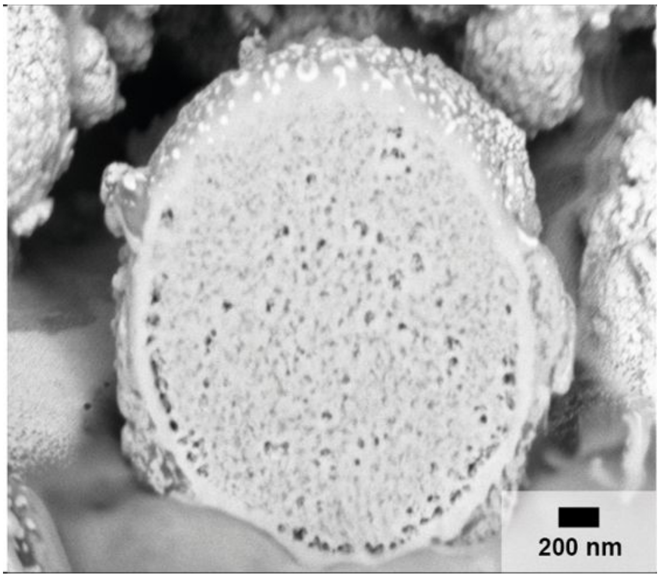

Figure S7: Focused ion beam milling-scanning electron microscopy (FIB-SEM) image of a cross section of the catalyst LaOCl:Nd/MAO/Cp_2ZrMe_2.

Different polymerization times:

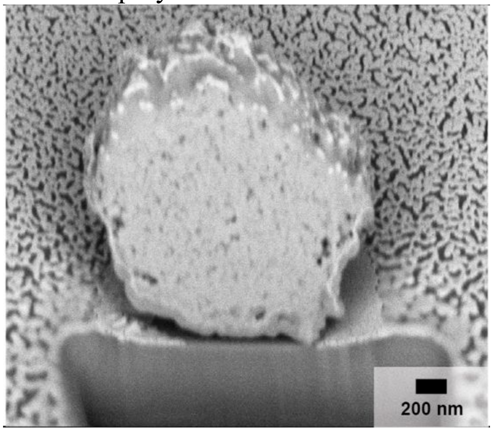

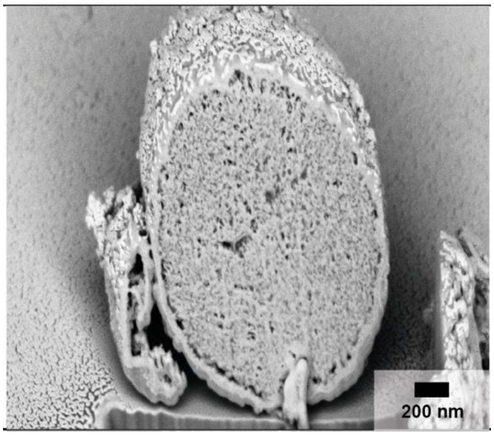

Figure S8: Focused ion beam milling-scanning electron microscopy (FIB-SEM) images of two cross sections of the ethylene polymerized LaOCl:Nd/MAO/Cp_2ZrMe_2 catalyst for 2 min, during the polymer shell formation phase, indicated in blue Figure 2 of the main text.

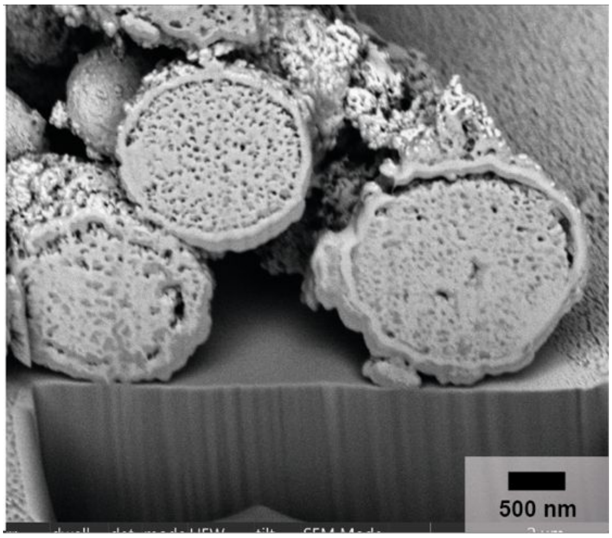

Figure S9: Focused ion beam milling-scanning electron microscopy (FIB-SEM) image of three cross sections of the ethylene polymerized LaOCl:Nd/MAO/Cp_2ZrMe_2 catalyst for 2:30 min, during the polymer shell break-up phase and first release of temperature, indicated in orange in Figure 2 of the main text.

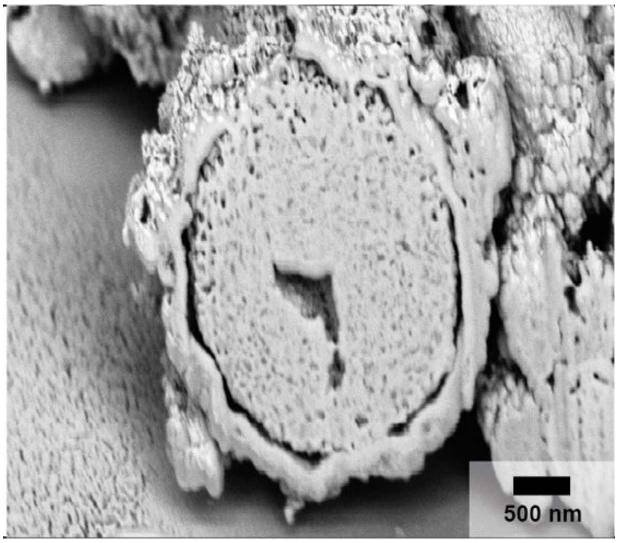

Figure S10: Focused ion beam milling-scanning electron microscopy (FIB-SEM) image of a cross section of the ethylene polymerized LaOCl:Nd/MAO/Cp_2ZrMe_2 catalyst for 3 min, during the fragmentation phase, indicated in green in Figure 2 of the main text.

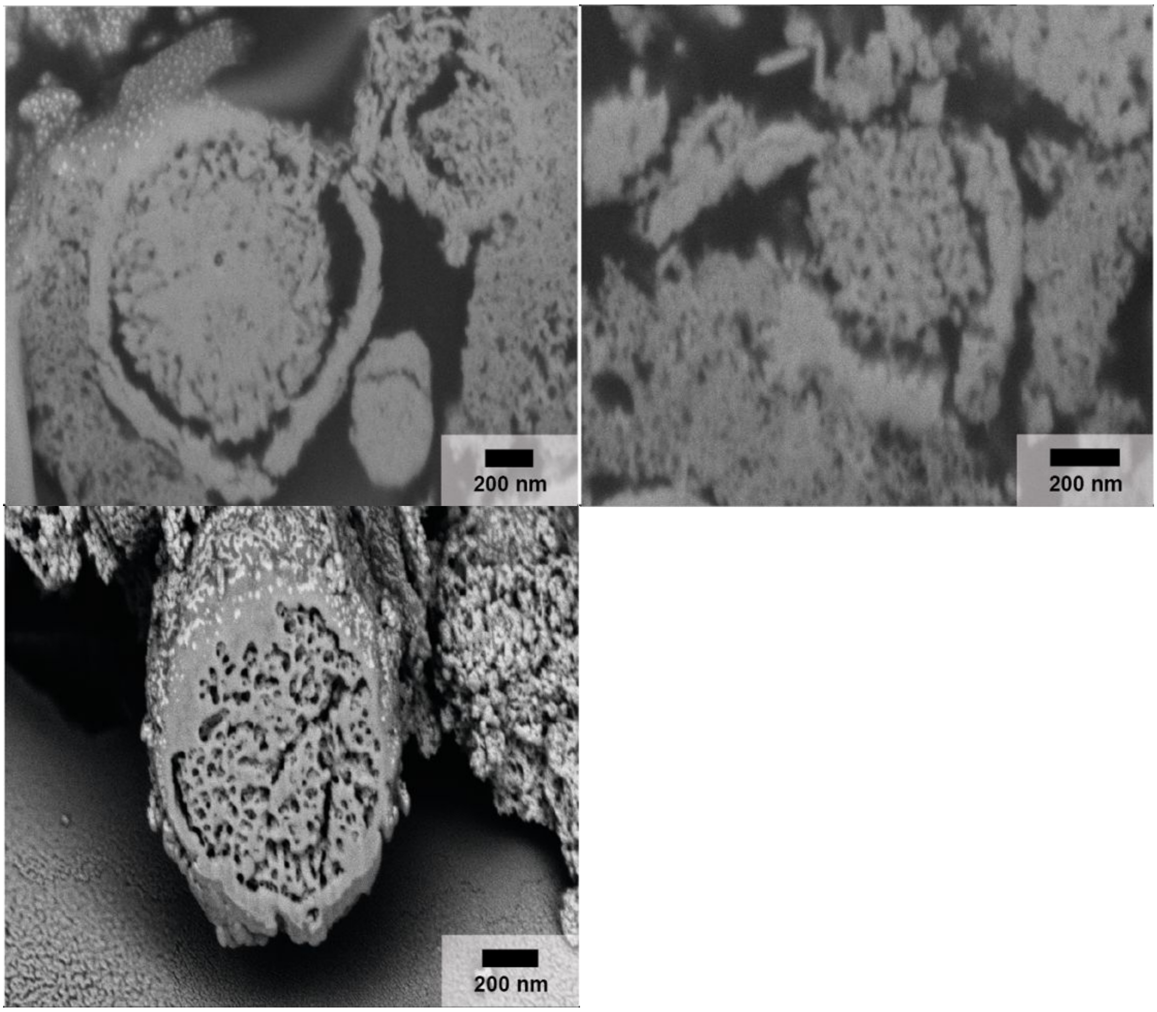

Figure S11: Focused ion beam milling-scanning electron microscopy (FIB-SEM) images of three cross sections of the ethylene polymerized LaOCl:Nd/MAO/Cp_2ZrMe_2 catalyst for 3:30 min, during the particle expansion and full fragmentation phase, indicated in red in Figure 2 of the main text.

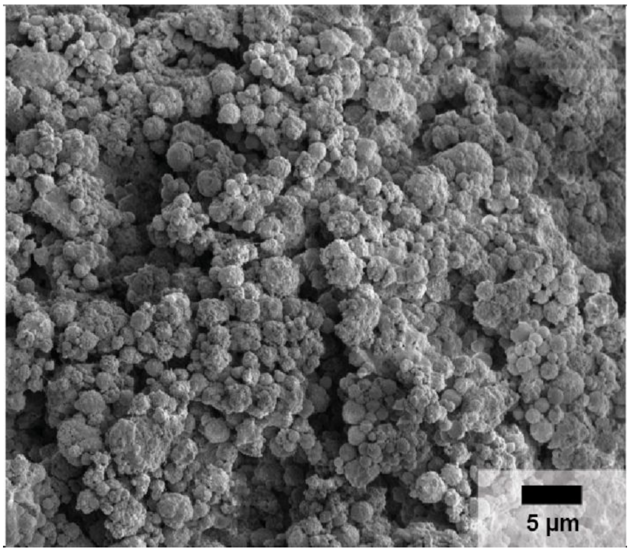

Figure S12: Scanning electron microscopy (SEM) image of the LaOCl:Nd/MAO/Cp_2ZrMe_2 catalyst ethylene polymerized for 60 minutes under mild gas phase conditions.

Final Polymer Slurry phase 60 minutes

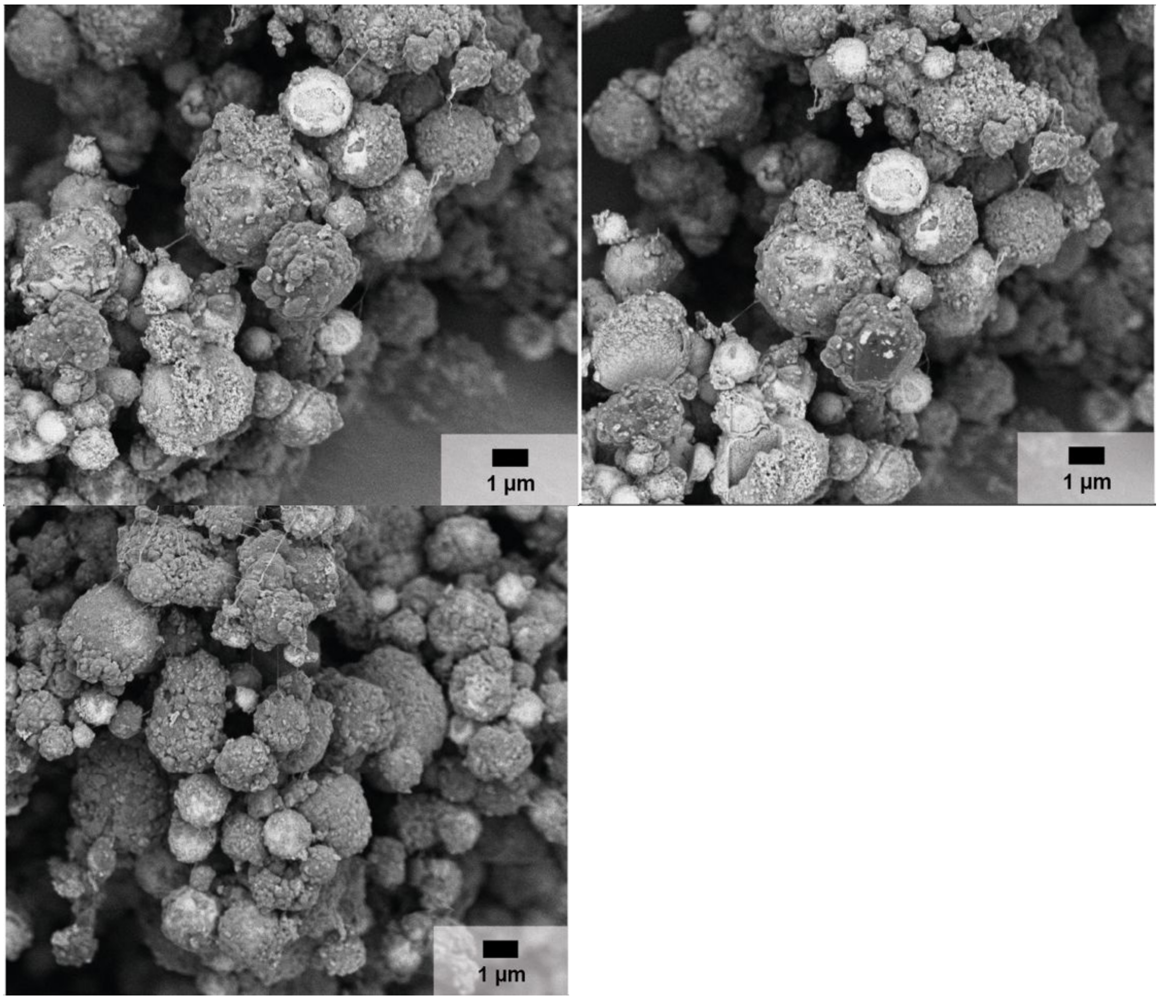

Figure S13: Focused ion beam milling-scanning electron microscopy (FIB-SEM) images of the LaOCl:Nd/MAO/Cp_2ZrMe_2 catalyst ethylene polymerized for 60 min under mild slurry phase conditions. Figure S12-2 is a cross section of Figure S12-1, indicating high heterogeneity present in the catalysts when polymerized.

4c Scanning electron microscopy-energy dispersive X-ray spectroscopy

EDX taken of the samples on silicon wafers and on carbon tape, to show C, Al & La distribution for the polymerized samples, and for the catalyst the Al & La distribution.

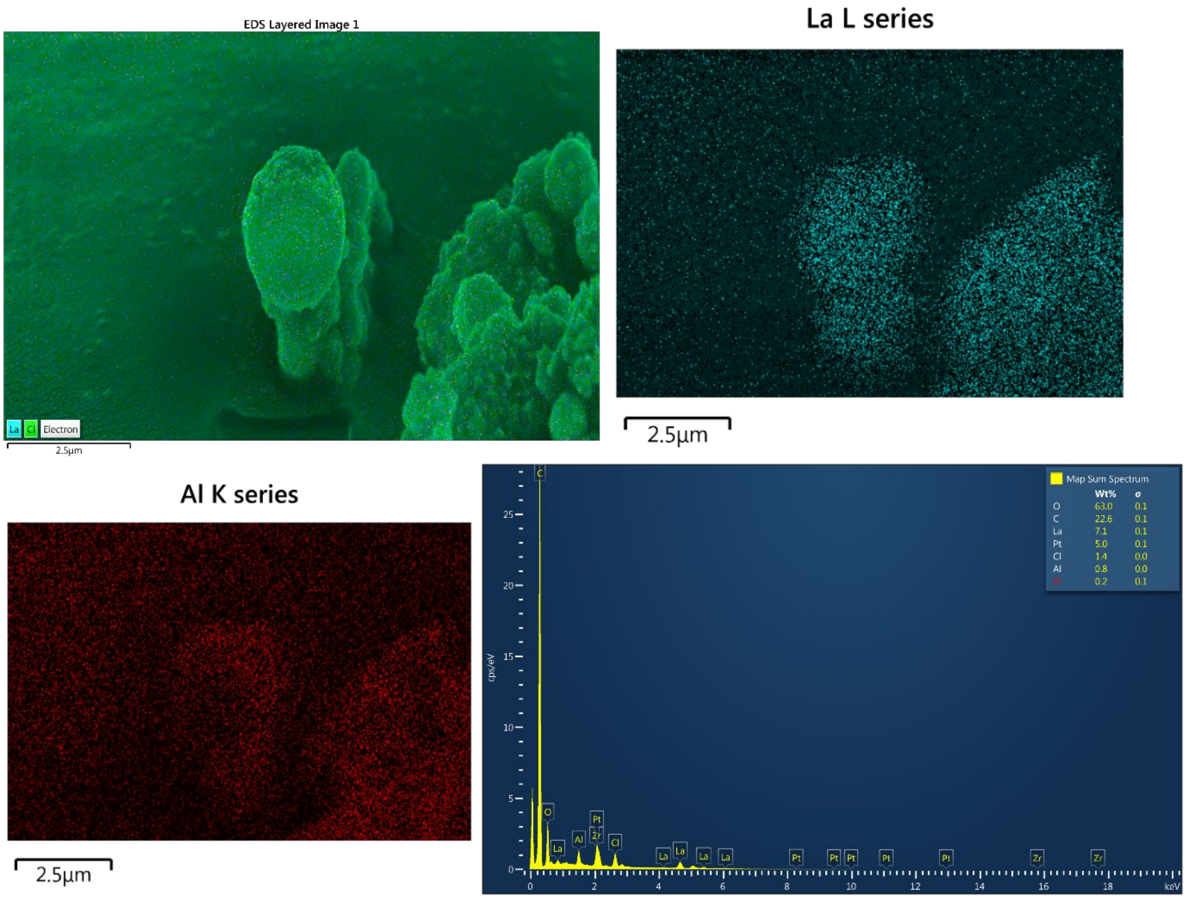

Figure S14: Scanning electron microscopy (SEM) image of the cross sectioned LaOCl:Nd/MAO/Cp_2ZrMe_2 catalyst on carbon tape with overlay. Respective La and Al distribution with corresponding energy dispersive X-ray (EDX) spectrum and elemental wt.%.

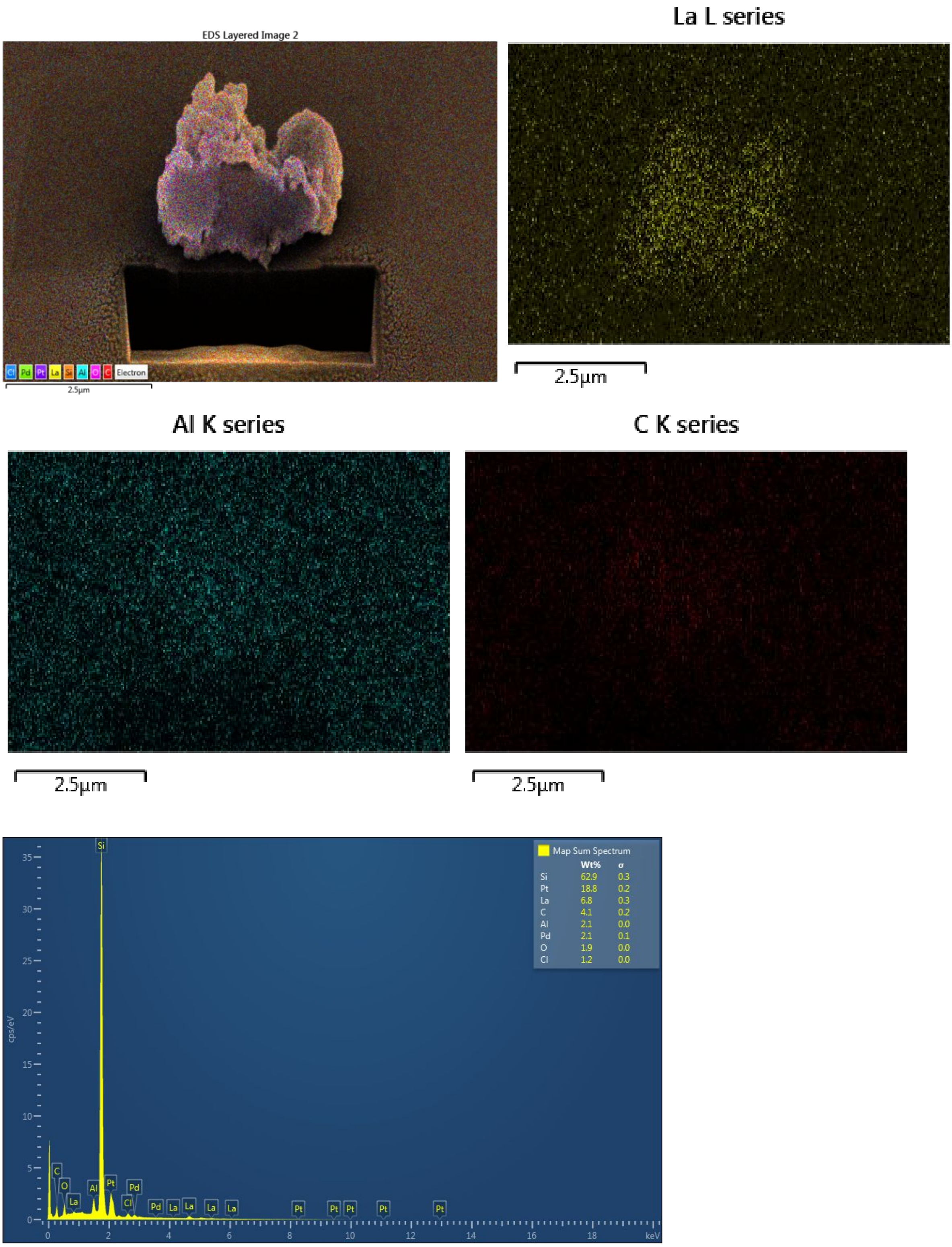

Figure S15: Scanning electron microscopy (SEM) image of the cross sectioned LaOCl:Nd/MAO/Cp_2ZrMe_2 catalyst polymerized for 2 min on a silicon wafer with overlay. Respective Al, La and C distribution with corresponding energy dispersive X-ray (EDX) spectrum and elemental wt.%.

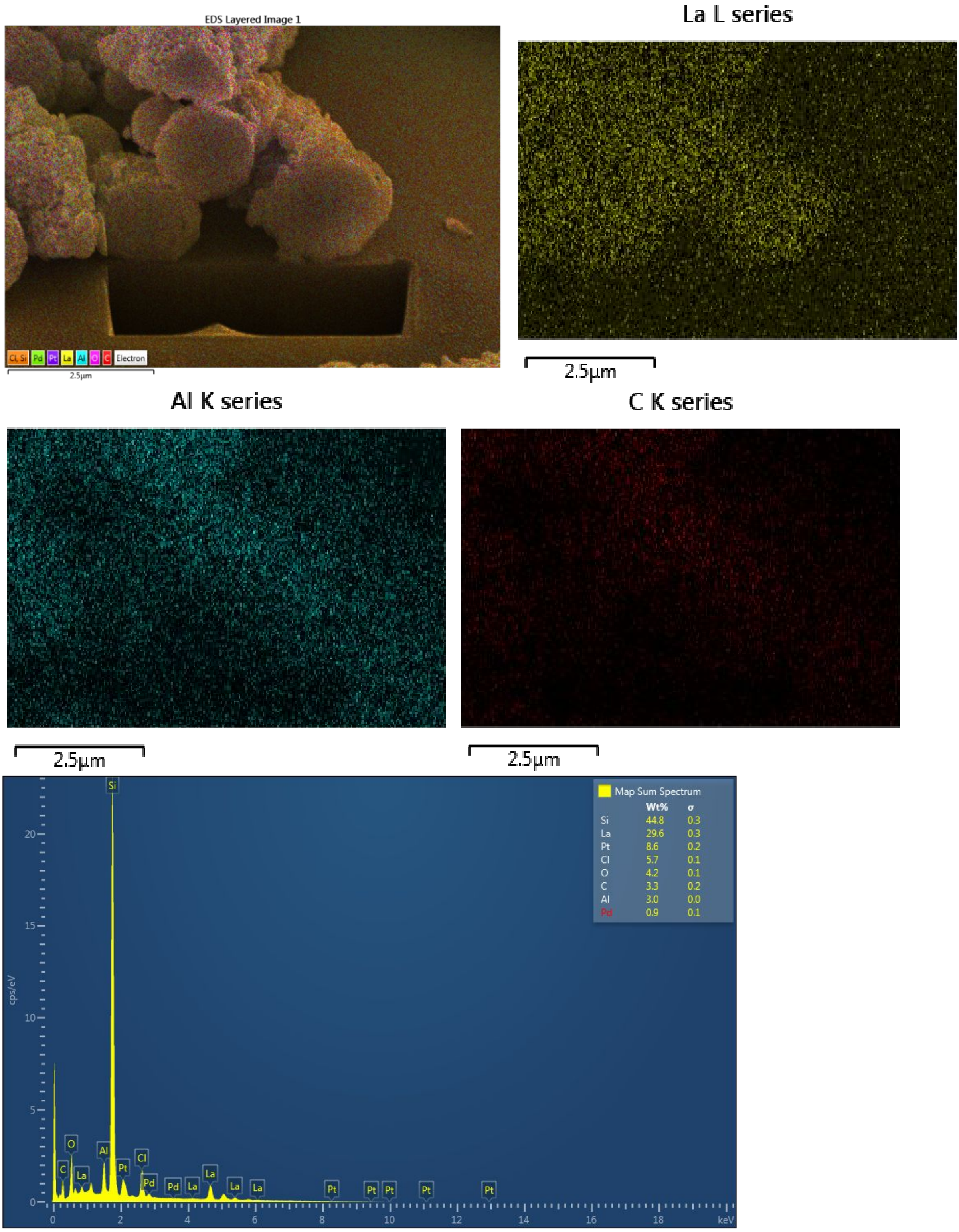

Figure S16: Scanning electron microscopy (SEM) image of the cross sectioned LaOCl:Nd/MAO/Cp_2ZrMe_2 catalyst polymerized for 2 min and 30 s on a silicon wafer with overlay. Respective Al, La and C distribution with corresponding energy dispersive X-ray (EDX) spectrum and elemental wt.%.

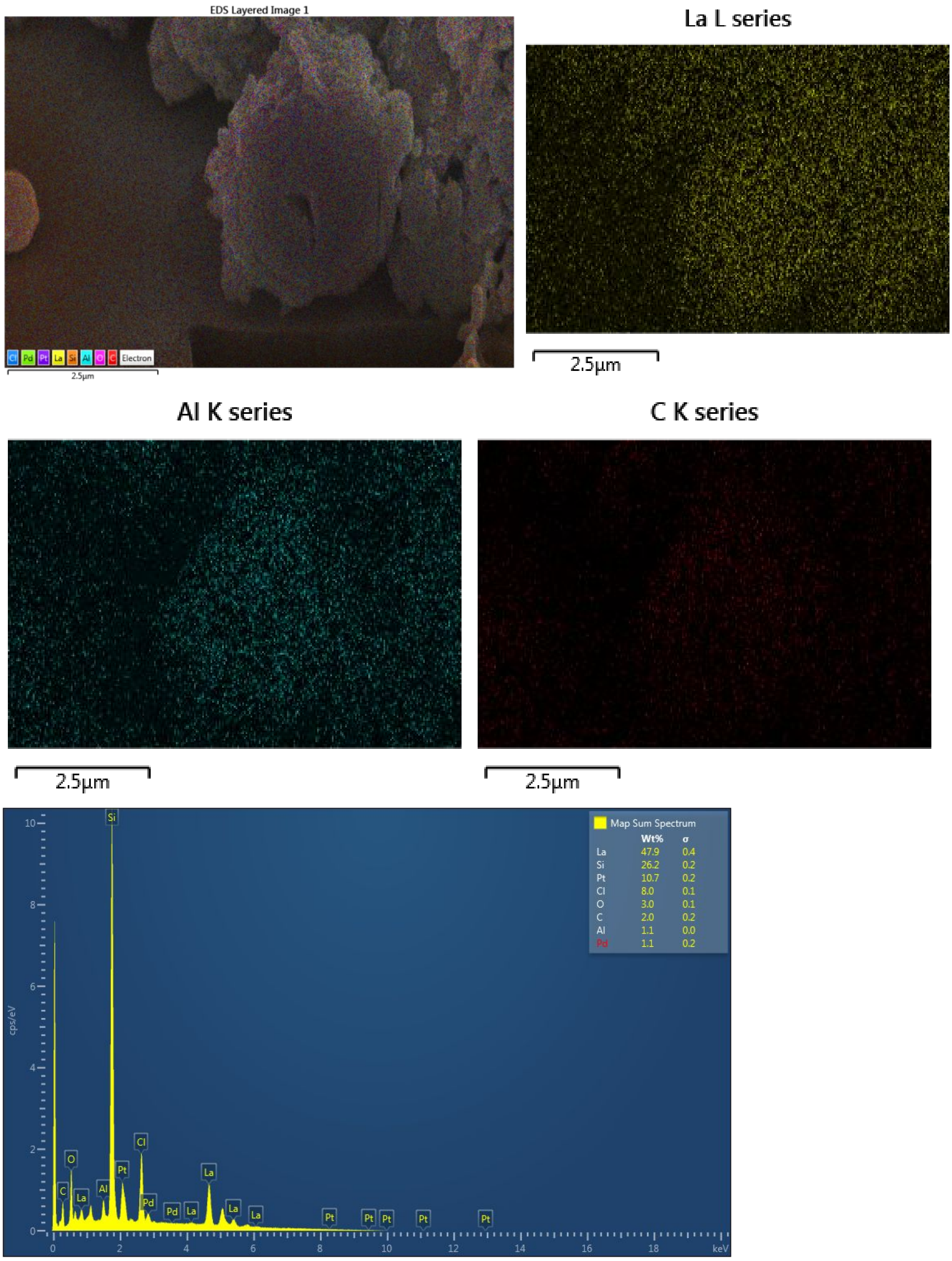

Figure S17: Scanning electron microscopy (SEM0 image of the cross sectioned LaOCl:Nd/MAO/Cp_2ZrMe_2 catalyst polymerized for 3 min on a silicon wafer with overlay. Respective Al, La and C distribution with corresponding energy dispersive X-ray (EDX) spectrum and elemental wt.%.

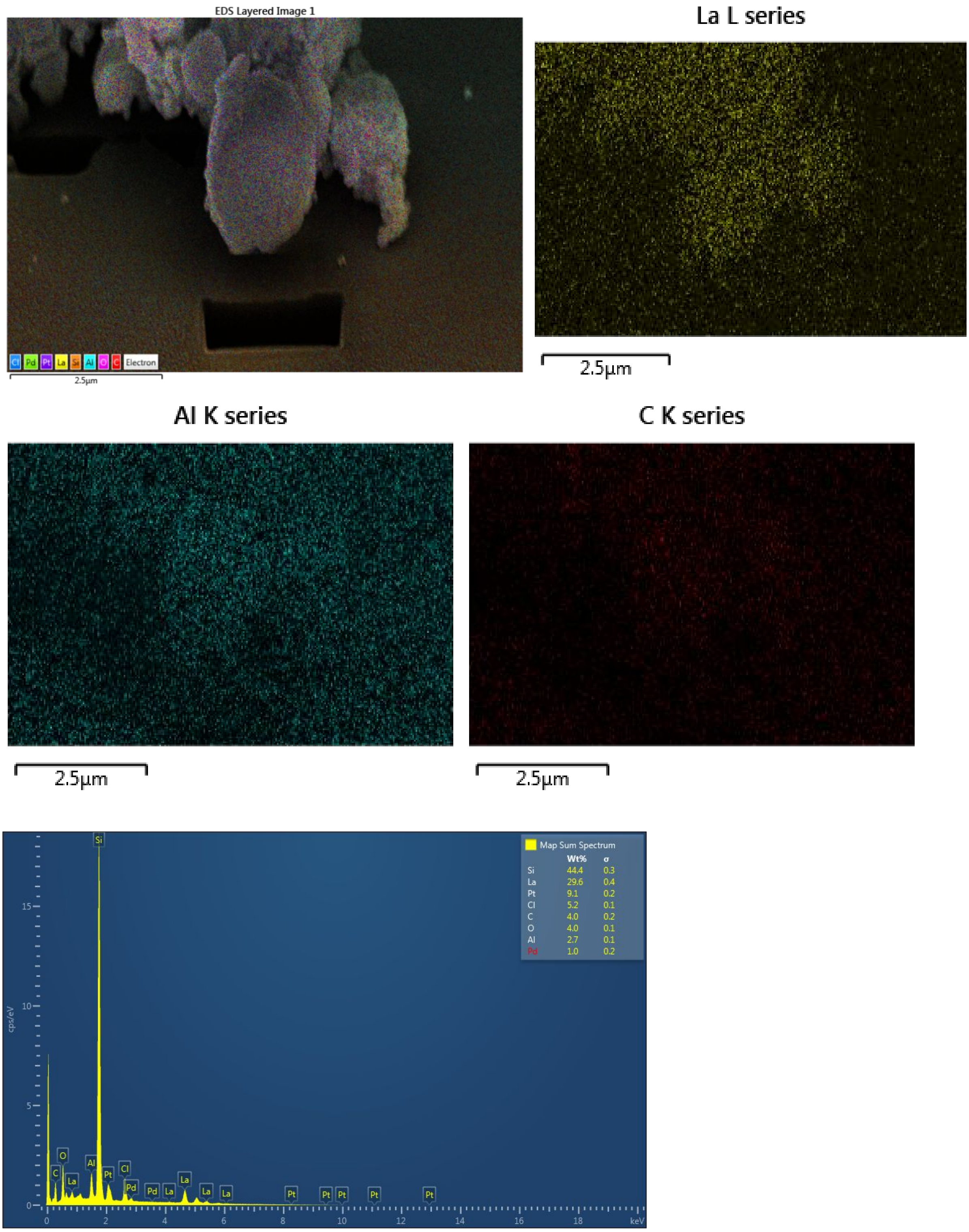

Figure S18: Scanning electron microscopy (SEM) image of the cross sectioned LaOCl:Nd/MAO/Cp_2ZrMe_2 catalyst polymerized for 3 min and 30 s on a silicon wafer with overlay. Respective Al, La and C distribution with corresponding energy dispersive X-ray (EDX) spectrum and elemental wt.%.

4d Physisorption data of the three samples (Adsorption isotherms, BJH adsorption & BJH desorption):

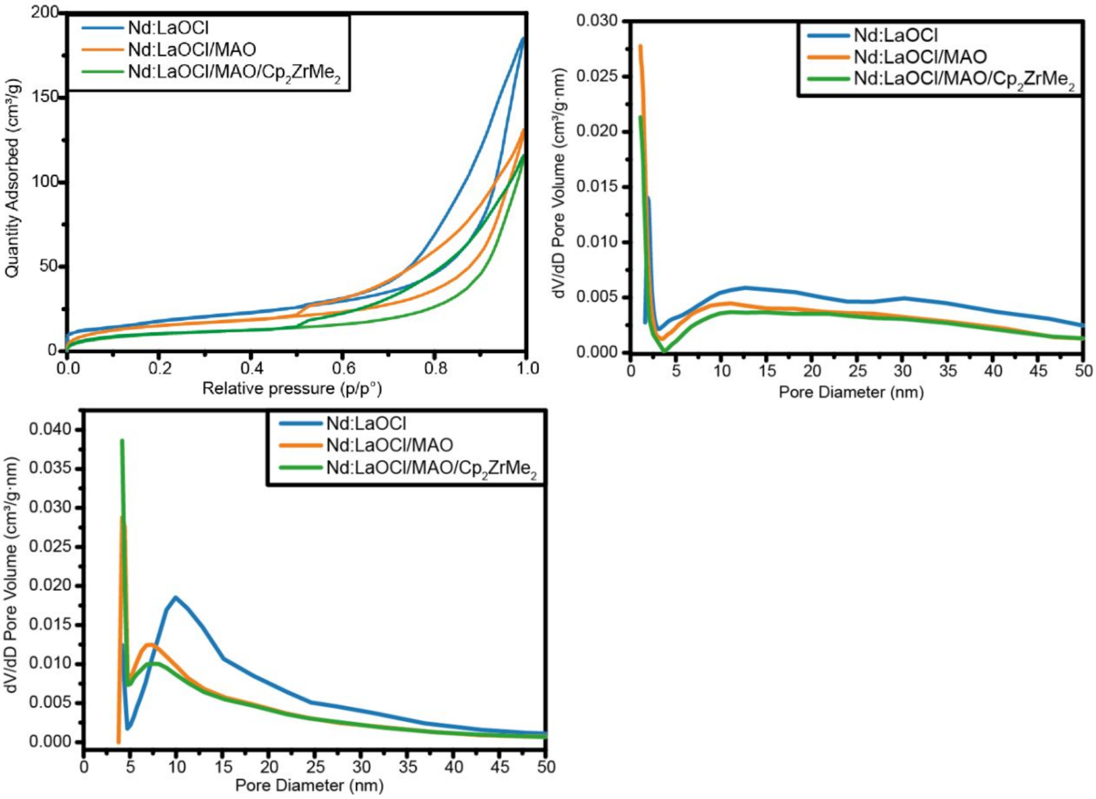

Figure S19: Physisorption, Brunauer-Joyner-Halenda (BJH) adsorption and BJH desorption isotherms of the support in blue (Nd:LaOCl), activated support in orange (LaOCl:Nd/MAO) and catalyst in green (LaOCl:Nd/MAO/Cp_2ZrMe_2).

Table S1: physical properties (surface area, pore volume and average pore diameter) determined by N_2 physisorption for support (Nd:LaOCl), activated support (LaOCl:Nd/MAO) and catalyst (LaOCl:Nd/MAO/Cp_2ZrMe_2).

Sample	LaOCl:Nd	LaOCl:Nd/MAO	LaOCl:Nd/MAO/Cp_2ZrMe_2
Surface area (m²/g)	69.06	55.87	37.88
Pore Volume (cm³/g)	0.29	0.20	0.18
Average Pore Diameter* (nm)	14.69	11.34	12.17

*= Calculated from BJH Desorption

4e. ICP-OES data of the catalyst/support matrix

Table S2: Inductively coupled plasma-optical emission spectroscopy (ICP-OES) elemental concentration values of 1 the support (Nd:LaOCl), 2 the activated support (LaOCl:Nd/MAO) and 3 the catalyst (LaOCl:Nd/MAO/Cp_2ZrMe_2)

**Sample**	**% La**	**% Nd**	**% Al**	**% Zr**	**Al/Zr**
**1 LaOCl:Nd**	98.1475506	1.849901011	0.002110144	0.000438215	-
**2 LaOCl:Nd/MAO**	77.5372499	1.499483332	20.96270358	0.000563158	-
**3 LaOCl:Nd/MAO/Cp_2ZrMe_2**	77.5818525	1.465103258	20.22226228	0.730781917	27.7

4f. X-ray diffraction

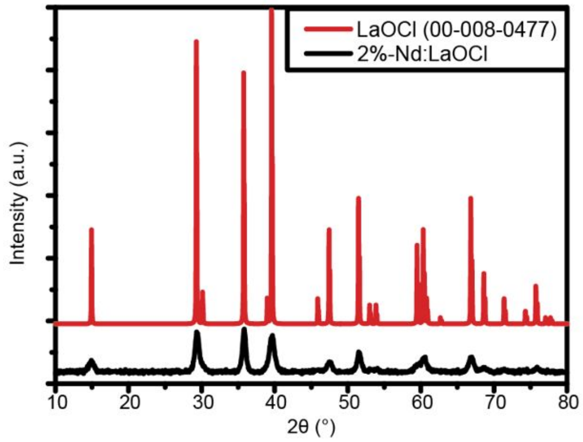

Figure S20: X-ray diffraction (XRD) patterns of reference LaOCl (00-008-0477) in red and the Nd: LaOCl support in black.

4g. Differential scanning calorimetry

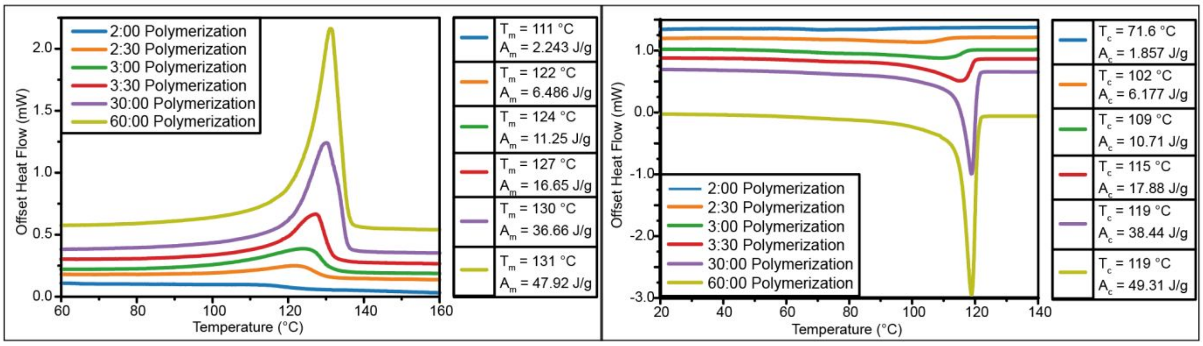

Figure S21: Differential scanning calorimetry (DSC) curves of the melting points (T_m) and crystallization temperature (T_c) of each polymerized sample with consecutive melting enthalpy, integrated melting peak (A_m) and crystallization enthalpy, integrated crystallization peak (A_c). Calculated crystallization of the samples in table below:

Table S3: Calculated crystallinity of the samples at different polymerization times under 1.2 ethylene bar pressure, room temperature gas-phase reaction.

**Sample**	**Crystallinity (X_c)***
2:00 polymerization	0.77%
2:30 polymerization	2.21%
3:00 polymerization	3.84%
3:30 polymerization	5.57%
30:00 polymerization	12.51%
60:00 polymerization	16.35%

*Degree of crystallinity (Xc) calculated by:[8] $X_c=\Delta H_m \cdot \Delta H^0_m \cdot 100\%$
With ΔH_m is measured heat of fusion and ΔH^0_m is the heat of fusion of 100% crystalline HDPE (293 J/g).[9]

5. Effect of reaction parameter changes in ultrasonic spray pyrolysis on LaOCl microspheres:

5a List of experiments

Table S4: List of experiments carried out to optimize the synthesis conditions of the LaOCl carrier.

Experiment	Temperature (°C)	Carrier Gas	Surfactant	Precursor ratio (surfactant: Salt)	Flowrate (ml/min)
**600-Air-C(1:10)-800**	600	air	CTAB	1:10	800
**700-Air-C(1:10)-800**	700	air	CTAB	1:10	800
**800-Air-C(1:10)-800**	800	air	CTAB	1:10	800
**900-Air-C(1:10)-800**	900	air	CTAB	1:10	800
**900-N_2-C(1:10)-800**	900	nitrogen	CTAB	1:10	800
**900-N_2-C(1:5)-800**	900	nitrogen	CTAB	1:5	800
**900-N_2-C(1:1)-800**	900	nitrogen	CTAB	1:1	800
**900-N_2-C(0:1)-800**	900	nitrogen	-	0:1	800
**900-N_2-C(1:10)-1200**	900	nitrogen	CTAB	1:10	1200
**900-N_2-C(1:10)-1500**	900	nitrogen	CTAB	1:10	1500
**700-Air-P(1:20)-800**	700	air	P-123	1:20	800
**700-Air-P(1:8)-800**	700	air	P-123	1:8	800
**700-Air-P(1:4)-800**	700	air	P-123	1:4	800

5b Effect of CTAB concentration

Table S5: List of experiments that were carried out to study the influence of CTAB concentration on the physical properties of LaOCl.

Experiment	S.A. (m^2/g)	V_{Pore} (cm^3/g)	Particle size (µm)
**900-N_2-C(0:1)-800**	23.9	0.12	0.98 ± 0.4
**900-N_2-C(1:10)-800**	28.3	0.21	1.10 ± 0.4
**900-N_2-C(1:5)-800**	18.5	0.10	1.12 ± 0.4
**900-N_2-C(1:1)-800**	24	0.07	-

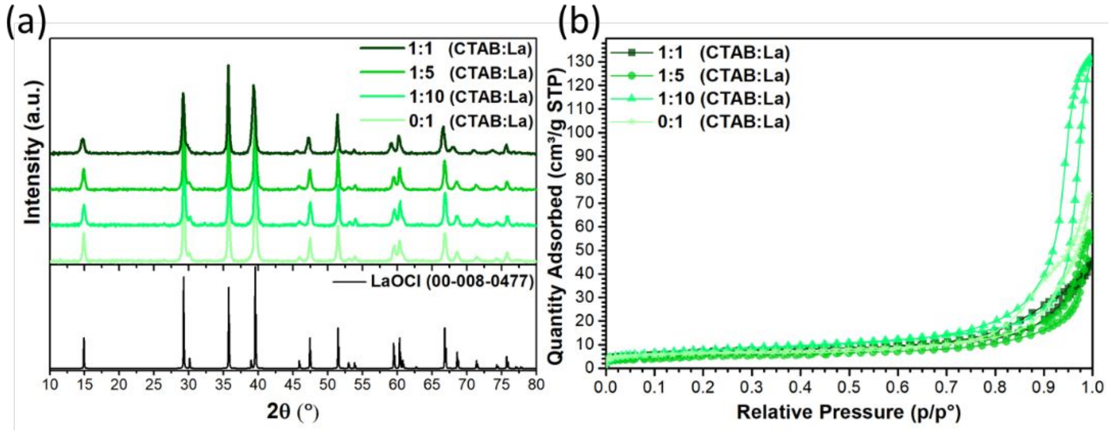

Figure S22: X-ray diffraction (XRD) patterns and N_2 physisorption isotherm of the LaOCl supports synthesized with different concentrations of cetrimonium bromide (CTAB).

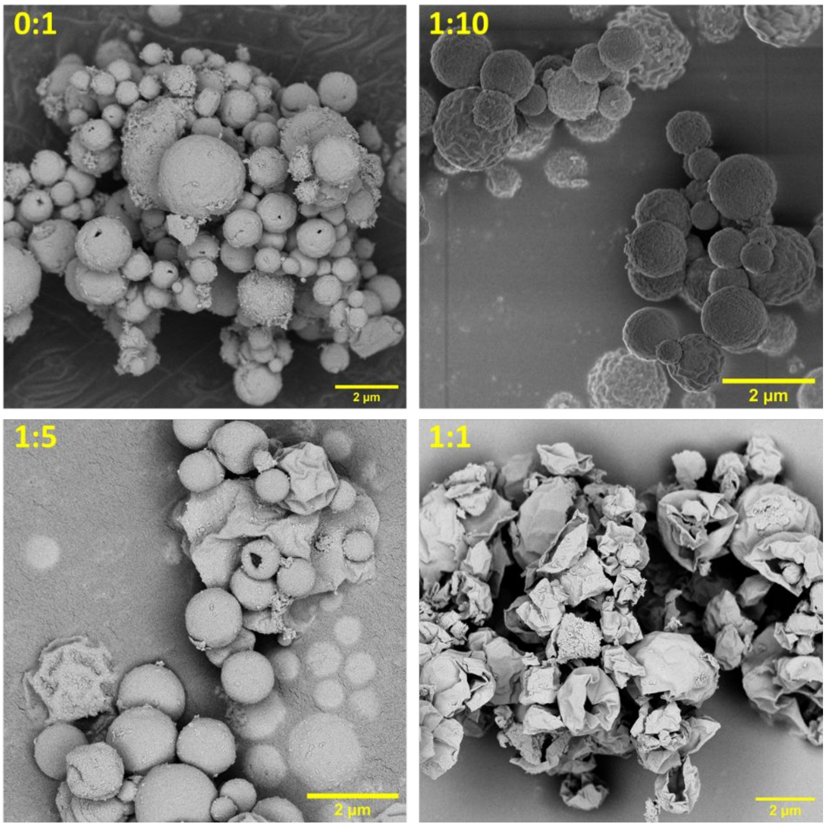

Figure S23: Scanning electron microscopy (SEM) images of the LaOCl supports synthesized with different concentrations of CTAB.

5c Effect of carrier gas flow:

Table S6: List of experiments that were carried out to study gas flow on the physical properties of LaOCl.

Experiment	S.A. (m^2/g)	V_{Pore} (cm^3/g)	Particle size (µm)
**900-N_2-C(1:10)-800**	28.3	0.21	1.10 ± 0.4
**900-N_2-C(1:10)-1200**	30.2	0.17	1.04 ± 0.4
**900-N_2-C(1:10)-1500**	32.5	0.23	1.03 ± 0.5

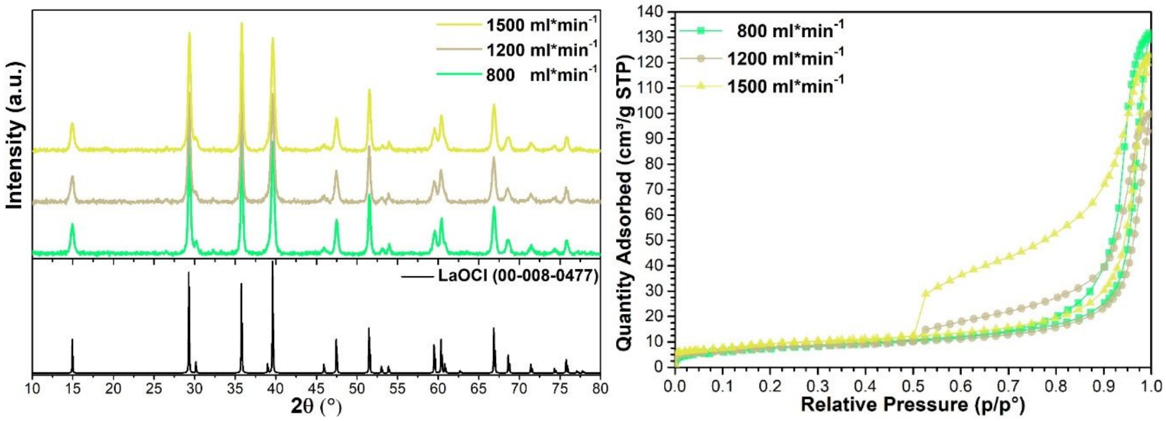

Figure S24: X-ray diffraction (XRD) patterns and N_2 physisorption isotherm of the LaOCl supports synthesized with different gas flows.

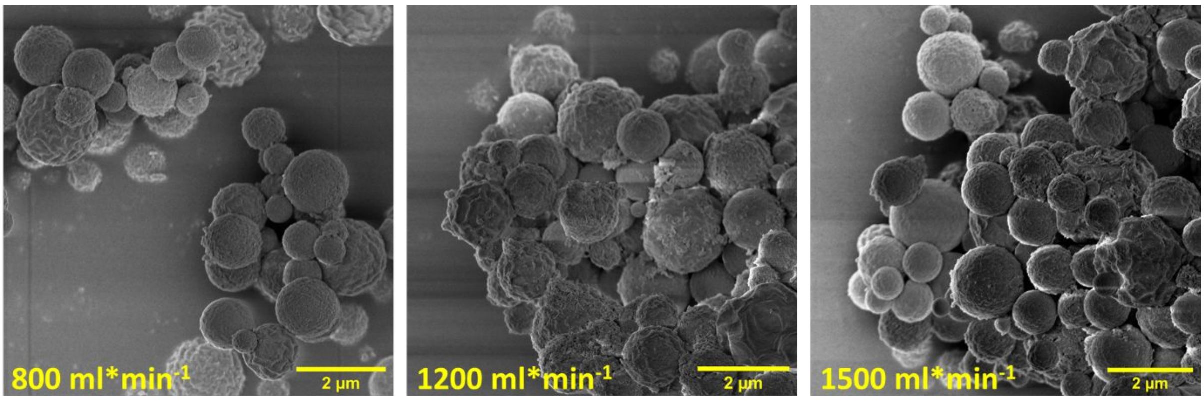

Figure S25: Scanning electron microscopy (SEM) images of the LaOCl supports synthesized with different gas flows.

5d Effect of carrier gas

Table S7: List of experiments that were carried out to study the effect of gas carrier on the physical properties of LaOCl.

Experiment	S.A. (m^2/g)	V_{Pore} (cm^3/g)	Particle size (µm)
**900-Air-C(1:10)-800**	40.6	0.28	1.05 ± 0.4
**900-N_2-C(1:10)-800**	28.3	0.21	1.10 ± 0.4

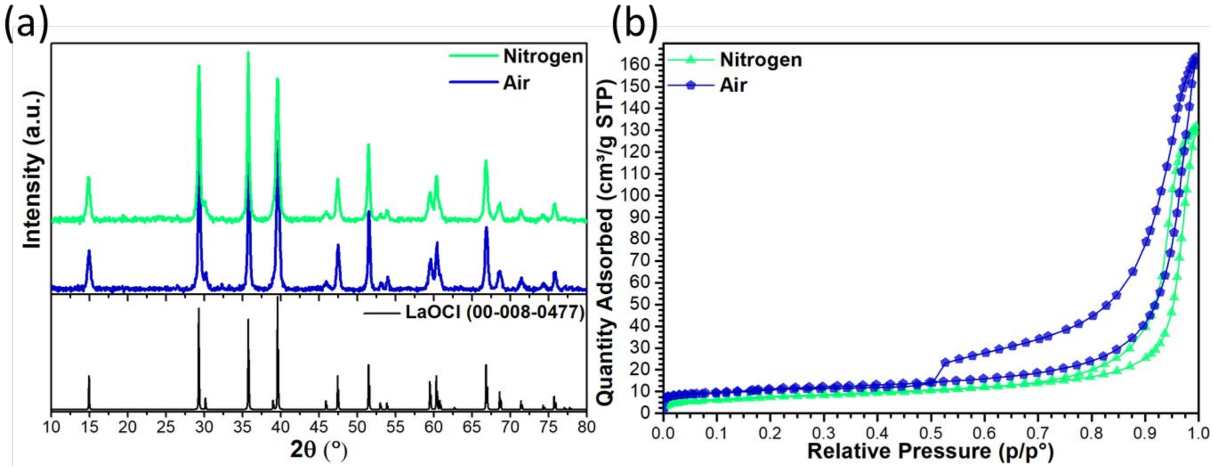

Figure S26: X-ray diffraction (XRD) patterns and N_2 physisorption isotherm of the LaOCl supports synthesized with different gas carriers.

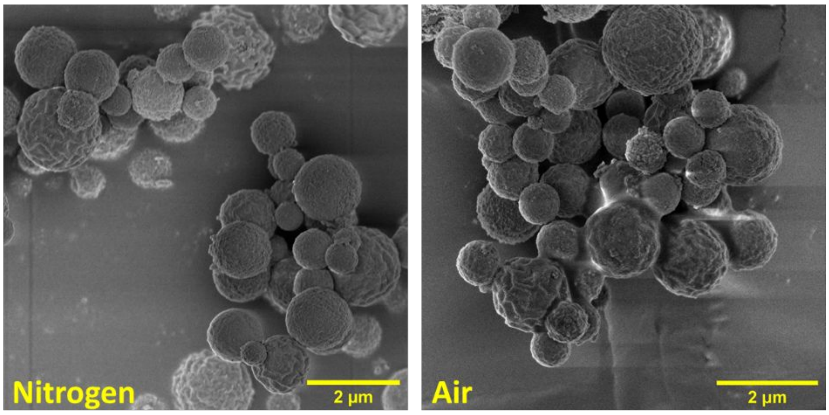

Figure S27: Scanning electron microscopy (SEM) images of the LaOCl supports synthesized with different gas carriers.

5e Effect of temperature

Table S8: List of experiments that were carried out to study the effect of oven temperature on the physical properties of LaOCl.

Experiment	S.A. (m^2/g)	V_{Pore} (cm^3/g)	Particle size (μm)
**600-Air-C(1:10)-800**	84.1	0.33	1.18 ± 0.5
**700-Air-C(1:10)-800**	78.0	0.31	1.06 ± 0.4
**800-Air-C(1:10)-800**	53.6	0.24	1.05 ± 0.5
**900-Air-C(1:10)-800**	40.6	0.28	1.05 ± 0.4

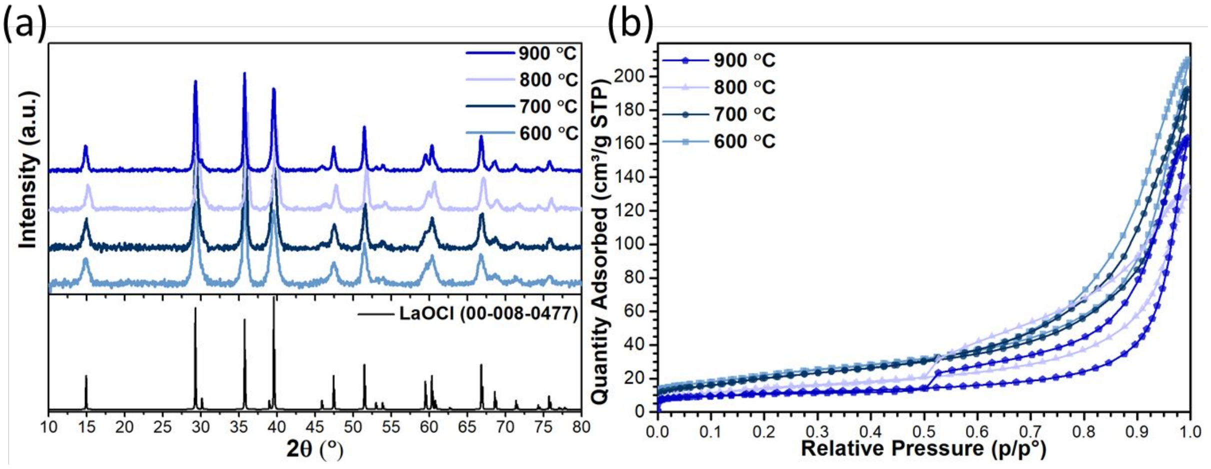

Figure S28: X-ray diffraction (XRD) patterns and N_2 physisorption isotherm of the LaOCl supports synthesized with different oven temperatures.

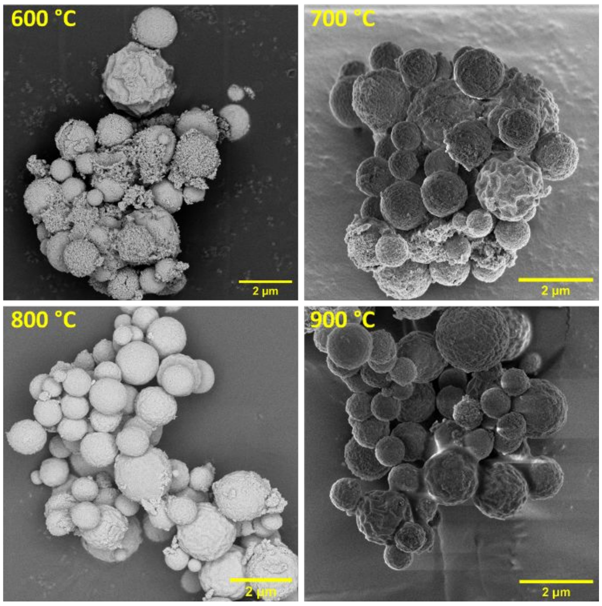

Figure S29: Scanning electron microscopy (SEM) images of the LaOCl supports synthesized with different oven temperatures.

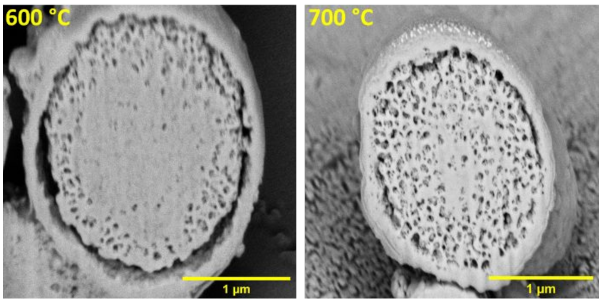

Figure S30: Focused ion beam-scanning electron microscopy (FIB-SEM) images of 2 cross sections of the LaOCl supports synthesized at 600 °C and 700 °C.

5f Effect of surfactant P123

Table S9: List of experiments that were carried out to study the effect of name (P123) concentration on the physical properties of LaOCl.

Experiment	S.A. (m^2/g)	V_{Pore} (cm^3/g)	Particle size (µm)
**700-Air-P(1:20)-800**	63.0	0.26	1.13 ± 0.5
**700-Air-P(1:8)-800**	64.3	0.33	1.31 ± 0.6
**700-Air-P(1:4)-800**	72.6	0.31	1.30 ± 0.5

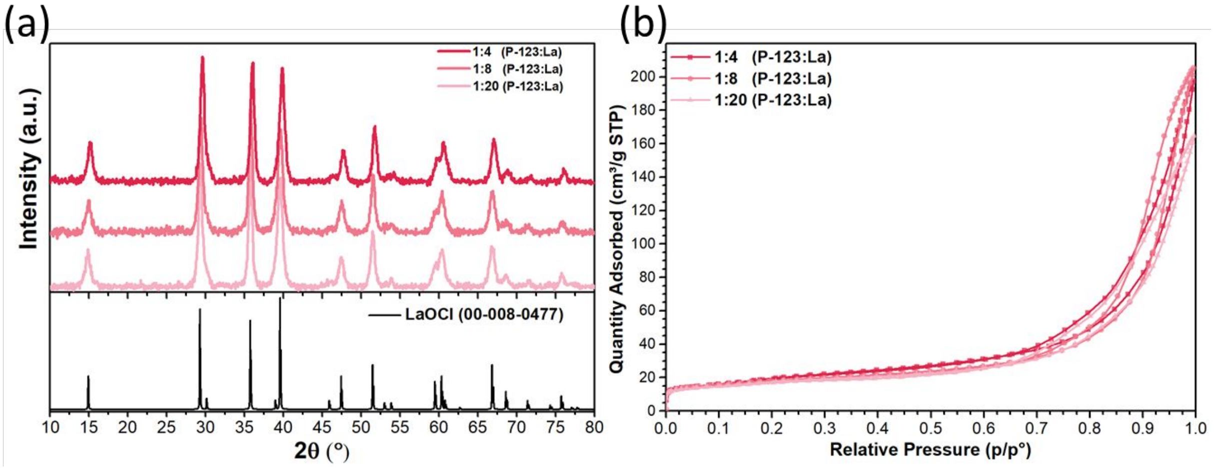

Figure S31: X-ray diffraction (XRD) diffractograms and N_2 physisorption isotherm of the LaOCl supports synthesized with different name (P123) concentrations.

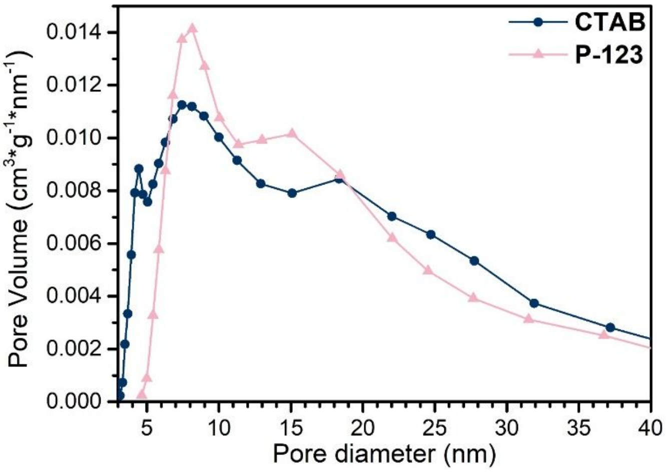

Figure S32: N_2 physisorption isotherm of a comparison between the pore structure of LaOCl synthesized with name (P123) and name (CTAB).

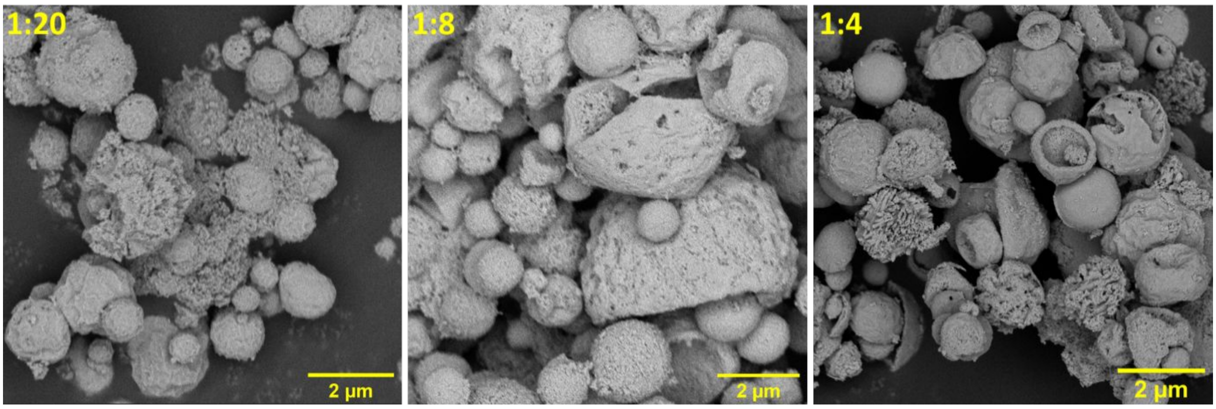

Figure S33: Scanning electron microscopy (SEM) images of LaOCl supports synthesized with different name (P123) concentrations.

5g Particle size distributions determined from scanning electron microscopy

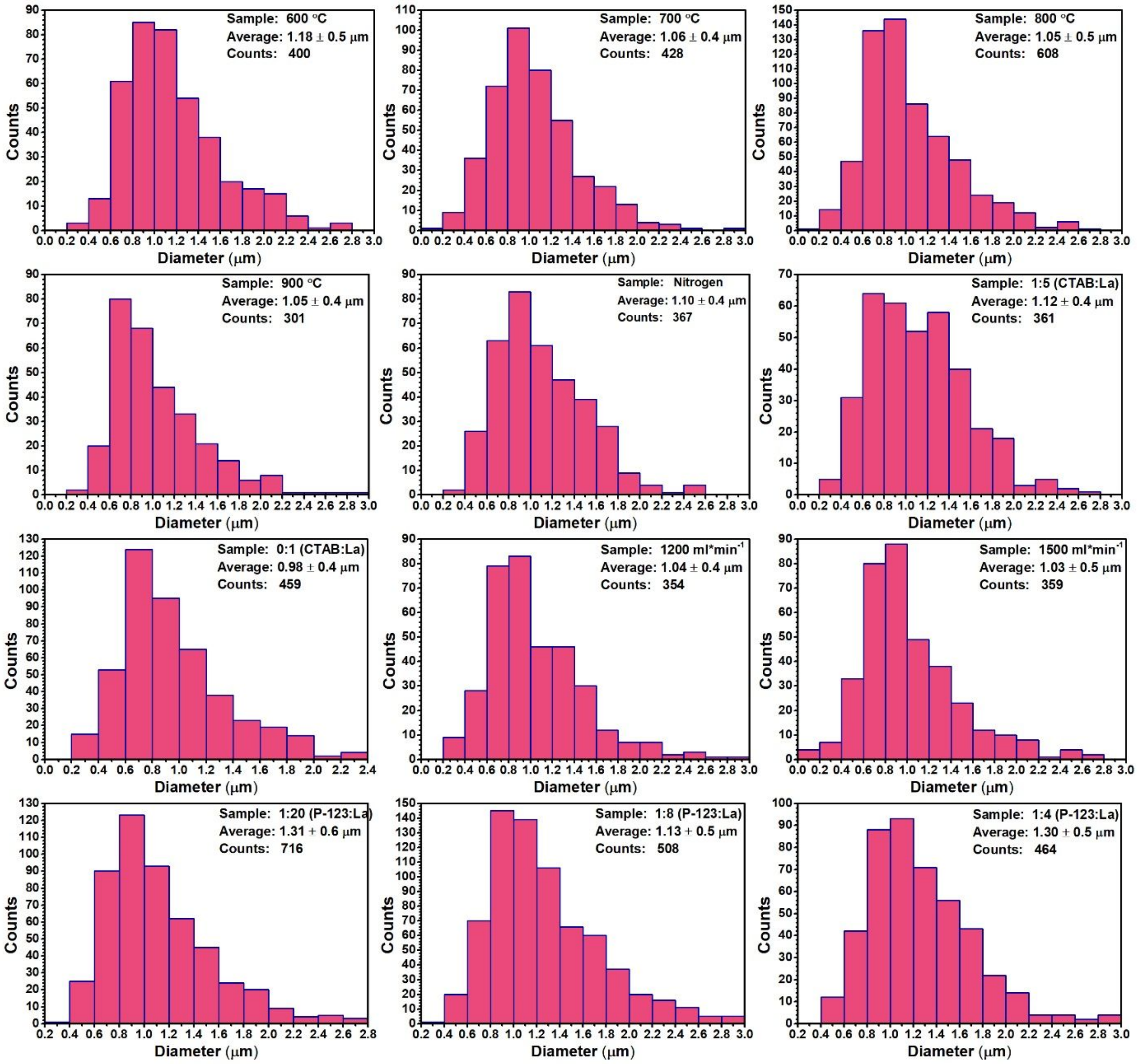

Figure S34: Particle size distributions of all synthesized samples of the LaOCl supports with different synthesis conditions.

6. References

(1) Zanoni, S.; Nikolopoulos, N.; Welle, A.; Vantomme, A.; Weckhuysen, B. M. Early-Stage Particle Fragmentation Behavior of a Commercial Silica-Supported Metallocene Catalyst. *Catal. Sci. Technol.* **2021**, *11* (15), 5335–5348. https://doi.org/10.1039/d1cy00930c.

(2) Mckenna, T. F. L.; Tioni, E.; Ranieri, M. M.; Alizadeh, A.; Boisson, C.; Monteil, V. Catalytic Olefin Polymerisation at Short Times: Studies Using Specially Adapted Reactors. *Can. J. Chem. Eng.* **2013**, *91* (4), 669–686. https://doi.org/10.1002/cjce.21684.

(3) Di Martino, A.; Broyer, J. P.; Spitz, R.; Weickert, G.; McKenna, T. F. A Rapid Quenched-Flow Device for the Characterisation of the Nascent Polymerisation of Ethylene under Industrial Conditions. *Macromol. Rapid Commun.* **2005**, *26* (4), 215–220. https://doi.org/10.1002/marc.200400530.

(4) Vonk, S. J. W.; van Swieten, T. P.; Cocina, A.; Rabouw, F. T. Photonic Artifacts in Ratiometric Luminescence Nanothermometry. *Nano Lett.* **2023**, *23* (14), 6560–6566. https://doi.org/10.1021/acs.nanolett.3c01602.

(5) van Swieten, T. P.; van Omme, T.; van den Heuvel, D. J.; Vonk, S. J. W.; Spruit, R. G.; Meirer, F.; Garza, H. H. P.; Weckhuysen, B. M.; Meijerink, A.; Rabouw, F. T.; Geitenbeek, R. G. Mapping Elevated Temperatures with a Micrometer Resolution Using the Luminescence of Chemically Stable Upconversion Nanoparticles. *ACS Appl. Nano Mater.* **2021**, *4* (4), 4208–4215. https://doi.org/10.1021/acsanm.1c00657.

(6) van Swieten, T. P.; Steenhoff, J. M.; Vlasblom, A.; de Berg, R.; Mattern, S. P.; Rabouw, F. T.; Suta, M.; Meijerink, A. Extending the Dynamic Temperature Range of Boltzmann Thermometers. *Light Sci. Appl.* **2022**, *11* (1), 343. https://doi.org/10.1038/s41377-022-01028-8.

(7) Geitenbeek, R. G.; De Wijn, H. W.; Meijerink, A. Non-Boltzmann Luminescence in NayF4:Eu3+: Implications for Luminescence Thermometry. *Phys. Rev. Appl.* **2018**, *10* (6), 064006. https://doi.org/10.1103/PhysRevApplied.10.064006.

(8) Tarani, E.; Arvanitidis, I.; Christofilos, D.; Bikiaris, D. N.; Chrissafis, K.; Vourlias, G. Calculation of the Degree of Crystallinity of HDPE/GNPs Nanocomposites by Using Various Experimental Techniques: A Comparative Study. *J. Mater. Sci.* **2023**, *58* (4), 1621–1639. https://doi.org/10.1007/s10853-022-08125-4.

(9) Wunderlich, B.; Czornyj, G. A Study of Equilibrium Melting of Polyethylene. *Macromolecules* **1977**, *10* (5), 906–913. https://doi.org/10.1021/ma60059a006.
